# Combining Mutations That Inhibit Two Distinct Steps of the ATP Hydrolysis Cycle Restores Wild-Type Function in the Lipopolysaccharide Transporter and Shows that ATP Binding Triggers Transport

**DOI:** 10.1128/mBio.01931-19

**Published:** 2019-08-20

**Authors:** Brent W. Simpson, Karanbir S. Pahil, Tristan W. Owens, Emily A. Lundstedt, Rebecca M. Davis, Daniel Kahne, Natividad Ruiz

**Affiliations:** a Department of Microbiology, The Ohio State University, Columbus, Ohio, USA; b Department of Chemistry and Chemical Biology, Harvard University, Cambridge, Massachusetts, USA; c Department of Biological Chemistry and Molecular Pharmacology, Harvard Medical School, Boston, Massachusetts, USA; University of Michigan—Ann Arbor; University of Milan; Genentech, Inc

**Keywords:** ABC transporters, ATPase, cell envelope, lipopolysaccharide, outer membrane

## Abstract

ATP-binding cassette (ABC) transporters constitute a large family of proteins present in all domains of life. They are powered by dynamic ATPases that harness energy from binding and hydrolyzing ATP through a cycle that involves the closing and reopening of their two ATP-binding domains. The LptB_2_FGC exporter is an essential ABC transporter that assembles lipopolysaccharides (LPS) on the surface of Gram-negative bacteria to form a permeability barrier against many antibiotics. LptB_2_FGC extracts newly synthesized LPS molecules from the inner membrane and powers their transport across the periplasm and through the outer membrane. How LptB_2_FGC functions remains poorly understood. Here, we show that the C-terminal domain of the dimeric LptB ATPase is essential for LPS transport in Escherichia coli. Specific changes in the C-terminal domain of LptB cause LPS transport defects that can be repaired by intragenic suppressors altering the ATP-binding domains. Surprisingly, we found that each of two lethal changes in the ATP-binding and C-terminal domains of LptB, when present in combined form, suppressed the defects associated with the other to restore LPS transport to wild-type levels both *in vivo* and *in vitro*. We present biochemical evidence explaining the effect that each of these mutations has on LptB function and how the observed cosuppression results from the opposing lethal effects these changes have on the dimerization state of the LptB ATPase. We therefore propose that these sites modulate the closing and reopening of the LptB dimer, providing insight into how the LptB_2_FGC transporter cycles to export LPS to the cell surface and how to inhibit this essential envelope biogenesis process.

## Abstract (precis)

Gram-negative bacteria are naturally resistant to many antibiotics because their surface is covered by the glycolipid LPS. Newly synthesized LPS is transported across the cell envelope by the multiprotein Lpt machinery, which includes LptB_2_FGC, an unusual ABC transporter that extracts LPS from the inner membrane. Like in other ABC transporters, the LptB_2_FGC transport cycle is driven by the cyclical conformational changes that a cytoplasmic, dimeric ATPase, LptB, undergoes when binding and hydrolyzing ATP. How these conformational changes are controlled in ABC transporters is poorly understood. Here, we identified two lethal changes in LptB that, when combined, remarkably restore wild-type transport function. Biochemical studies revealed that the two changes affect different steps in the transport cycle, having opposing, lethal effects on LptB’s dimerization cycle. Our work provides mechanistic details about the LptB_2_FGC extractor that could be used to develop Lpt inhibitors that would overcome the innate antibiotic resistance of Gram-negative bacteria.

## INTRODUCTION

The outer membrane (OM) of most Gram-negative bacteria is covered with lipopolysaccharide (LPS) molecules that create a permeability barrier against many antimicrobials (1). Since LPS biogenesis is also essential for the viability of important pathogens (2, 3), it is an attractive target for developing new molecules that might kill Gram-negative bacteria or might at least permeabilize their OM to antibiotics that otherwise cannot efficiently cross the LPS barrier. LPS biogenesis encompasses three main pathways: (i) LPS biosynthesis starting in the cytoplasm and ending in the inner membrane (IM), (ii) transport of precursors across the IM, and (iii) transport of fully synthesized LPS molecules from the IM to the OM (4). Compounds that target each of these three processes have been reported previously and are being developed as novel antibiotics that prevent LPS assembly at the cell surface (reviewed in reference 5). Excitingly, a peptidomimetic that inhibits LPS transport from the IM to the OM in Pseudomonas aeruginosa is undergoing clinical trials (6–8).

**FIG 1:**
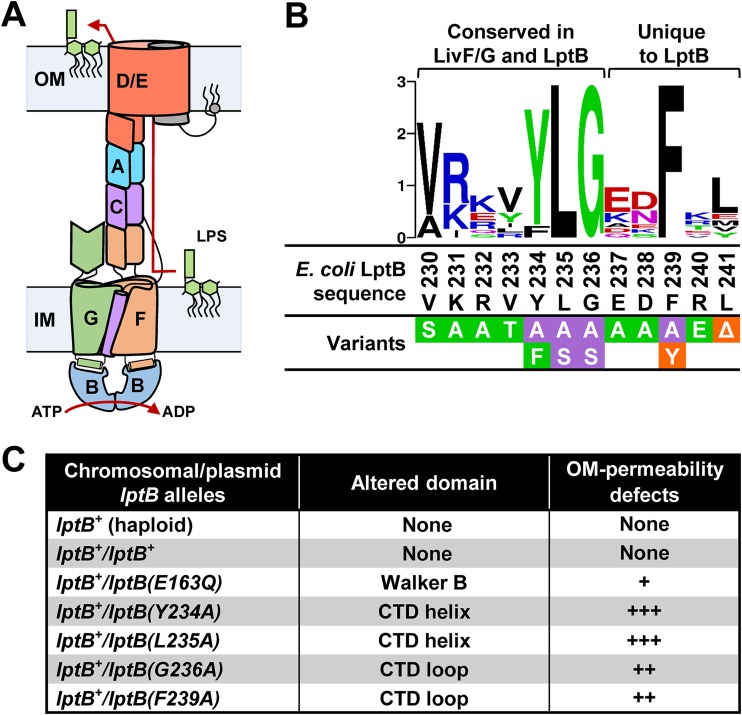
The CTD of LptB is conserved and essential for function. (A) Architecture of the Lpt transenvelope complex. (B) Sequence logo of the CTD of LptB generated with WebLogo software (weblogo.berkeley.edu) using an alignment of LptB homologs (44). Regions conserved in LivF/G homologs and unique to LptB homologs are marked above the logo. See alignment of LivF/G and LptB in Fig. S2. Below the logo, the sequence of the CTD from E. coli LptB^WT^ (residues 230 to 241) is shown in black. A summary of the results of functional analysis of variants with single amino acid substitutions in the CTD and encoded in pET23/42-LptB is shown. Substitutions conferring no defects in strains carrying a chromosomal Δ*lptB* deletion are shown in green boxes, those conferring a partial loss of function are shown in orange boxes, and those resulting in a total loss of function are shown in purple boxes. (C) Total-loss-of-function *lptB* alleles encoded in the pET23/42 plasmid confer dominant-negative effects, as determined by their ability to increase the OM permeability of merodiploid strains carrying the native chromosomal wild-type *lptB* allele (*lptB^+^*). The relative increase in OM permeability, which is indicative of LPS transport defects, is represented with plus signs (+). The catalytically defective LptB variant carrying the E163Q substitution in the Walker B domain was included for reference. Refer to Data set S1 for complete data and details.

Transport of LPS from the IM to the OM relies on Lpt (LPS transport) proteins LptA to LptG, which form a transenvelope machine that likely functions similarly to a Pez candy dispenser (Fig. 1A) (reviewed in reference 9). The IM Lpt components continuously extract newly synthesized LPS molecules from the bilayer and place them in the Lpt periplasmic bridge, creating a stream of LPS along the Lpt machine that is pushed from the base of the transporter at the IM toward the OM (9–11). Mechanistic details of this intermembrane LPS transporter remain largely unknown, but it is clear that it is powered by LptB_2_FGC, an unusual ABC transporter localized at the IM (12–14). Like other ABC transporters, LptB_2_FGC contains two nucleotide-binding domains (NBDs; LptB_2_) that function as a single ATPase that interacts with and powers two transmembrane domains (TMDs; LptF and LptG), which transport the LPS substrate (10, 15). However, unlike most ABC transporters, LptB_2_FGC does not translocate its substrate across the membrane; instead, it extracts LPS from the IM and places it onto a periplasmic protein bridge. Recent structural and biochemical studies have revealed unique structural features and key functional details of this transporter (16, 17). The six transmembrane helices of each of the two TMDs, LptF and LptG, associate with the single transmembrane helix of another protein, LptC, to form a cavity in the IM. LPS enters the cavity formed by the transmembrane helices of LptFGC in an ATP-independent manner. The cavity then undergoes a collapse that “squeezes” or “pumps” LPS out and places it onto the Lpt periplasmic bridge. The periplasmic domains of LptFGC each adopt a β-jelly roll fold, and those in LptF and LptC interact to provide a continuous path that LPS follows after exiting the LptFG cavity (Fig. 1A).

10.1128/mBio.01931-19.3 FIG S2 Sequence alignment of LptB and the ATPases of branched-chain amino acid transporters. Clustal W alignment of LptB homologs with closely related branched-chain amino acid transporter ATPases LivF/G shows that they share homology at their CTDs. The protein family PF12399 sequence is a conserved sequence found in both protein families and ending in a characteristic YLG sequence (1). The CTD of LptB has additional conserved residues not found in PF12399. Colors correspond to the percent identity color scheme in Jalview (2). Light to dark purple coloring indicates 50% to 100% identity, respectively, and white indicates <50% identity. The protein sequences aligned were those of LivF (WP_000416891.1), LivG (WP_136807488.1), and LptB (WP_000224099.1) from E. coli K-12 substrain MG1655 and LptB homologs from Geobacter uraniireducens Rfr and Burkholderia cenocepacia (WP_011939814.1 and WP_124633598.1, respectively). Download FIG S2, PDF file, 0.4 MB. Copyright © 2019 Simpson et al. 2019 Simpson et al. This content is distributed under the terms of the Creative Commons Attribution 4.0 International license.

10.1128/mBio.01931-19.9 DATA SET S1 (Page 2) Loss-of-function substitutions at the CTD of LptB confer dominant-negative phenotypes. (Page 3) OM permeability defects of haploid strains with mutations in *lptB* that disrupt the interaction between the switch helix and CTD loop. (Page 4) Genetic interactions between changes in R144 and R198 and alterations to the CTD of LptB. (Page 5) Genetic interactions between changes in Walker A residue T45 and alterations to the CTD of LptB. (Page 6) Strains used in this study. (Page 9) Primers used in this study. Download Data Set S1, PDF file, 0.3 MB. Copyright © 2019 Simpson et al. 2019 Simpson et al. This content is distributed under the terms of the Creative Commons Attribution 4.0 International license.

ABC transporters use ATP binding and hydrolysis to cycle between conformations that promote substrate binding, substrate translocation, and resetting of the transporter to its ground state (18–22). ATP binding drives the formation of a closed NBD dimer that sandwiches two ATP molecules, and then hydrolysis promotes dimer opening to allow ADP/ATP exchange. Since NBDs are physically connected to TMDs, the nucleotide-driven dynamics of the NBDs cause the TMDs to cycle between states. In some transporters, ATP binding directs substrate transport, while ATP hydrolysis and subsequent release of ADP and inorganic phosphate (P_i_) reset them to their ground state; in others, ATP hydrolysis powers substrate transport and the subsequent release of ADP and P_i_ resets them (19). Here, we discovered a novel C-terminal domain (CTD) in LptB that is crucial for LPS transport in Escherichia coli. Changes to this domain reduce ATP hydrolysis and prevent proper cycling of LptB. Our genetic, biochemical, and structural work provides insight into the LPS transport cycle by elucidating how different sites in LptB affect the function of this ATPase (summarized in Table 1).

**TABLE 1:** Relevant domains and LptB variants explored in this study

Domain altered	Domain function (reference[s])	LptB variant(s) explored^*a*^
Near/in Walker A	ATP binding (18, 29)	G33A/C, L35Q, T43S, T45A
Walker B	ATP hydrolysis (15, 18)	**E163Q**
D-loop helix	ATPase dimerization (41)	K177A/E, E199A
Signature helix	ATP binding (this study)	**R144H¸** R144A/F/Q, R145A, I148S/T, S172A, D175A, I176T
Switch helix	Interaction with CTD loop (this study)	R198A/E
CTD helix	Unknown essential role (this study)	Y234A, L235A
CTD loop	ATPase dimerization and turnover (this study)	**F239A**, G236A, L241Δ
Extensions added to CTD loop	ATPase dimerization and turnover (this study)	**LptB1**, **LptB-EHis_8_**, LptB-R, LptB-RHis8

a Variants that add extended sequences or tags to the CTD of LptB are indicated with the allele names shown in Fig. S1. Variants with amino acid substitutions are indicated with the substitution. The most extensively studied variants are shown in bold.

10.1128/mBio.01931-19.2 FIG S1 Altering the CTD of LptB results in functional defects. (A) The addition of CT extensions to LptB disrupts activity to differing degrees. A summary of functional analyses for various *lptB* haploid strains is presented. In investigating why some CT-tagged LptB variants could support growth while others could not, we noticed that the two types significantly differed at residue 242, which is located in the linker between the last residue (L241) of wild-type LptB and the tag. The lethal chromosomal *lptB-His* allele encodes a positively charged arginine, while the complementing plasmidborne *lptB-EHis_8_* and chromosomal *lptB1* alleles encode a negatively charged glutamate and a neutral isoleucine, respectively. To examine if the addition of an arginine after the native L241 residue affected LptB function on its own or in the context of the His tag, we constructed plasmids bearing *lptB* alleles encoding LptB followed by only an arginine (*lptB-R*) or an arginine followed by a polyhistidine tag (*lptB-RHis_8_*) and compared their function to that of wild-type *lptB* (*lptB^+^*) and *lptB-EHis_8_* genes. The *lptB-R* and *lptB-EHis_8_* alleles complemented chromosomal Δ*lptB*, but the resulting haploid strains were sensitive to hydrophobic antibiotics, indicating that these strains, like an *lptB1* strain, are defective in LPS transport. In contrast, the *lptB-RHis_8_* allele could not complement chromosomal Δ*lptB* despite resulting in the production of as much protein as an *lptB-EHis_8_* strain (see below). Thus, adding an arginine or oligopeptide to the CTD of LptB causes partial loss-of-function defects, while the combination of an arginine and oligopeptide is lethal. (B) LptB immunoblot comparing strains that chromosomally encode LptB^WT^, labeled 754, or LptB1. (C) LptB immunoblot of samples from haploid strains bearing LptB CTD variants on the pET23/42-LptB plasmid. “WT” refers to haploid strain NR2101, which produces LptB^WT^ from pET23/42-LptB. (D) LptB immunoblot of samples from merodiploid strains producing LptB CTD variants from the pET23/42-LptB plasmid and LptB^WT^ from the native *lptB* locus. “754” refers to NR754, the wild-type strain with *lptB^+^* at the native locus. “WT” refers to strain NR2583, which produces LptB^WT^ from both the chromosome and pET23/42-LptB. Data are representative of results from at least three independent experiments. (E) Ability of CTD *lptB* alleles to complement a chromosomal Δ*lptB* allele in rich (LB) medium. (F) OM permeability of haploid strains producing LptB CTD variants was determined by disc diffusion assay. All alleles, except *lptB^+^* in NR754 and *lptB1*, which are located on the chromosome, are borne on the pET23/42-LptB plasmid. Increased sensitivity was reproducibly observed for the strains shown and was indicative of LPS transport defects compared to wild-type *lptB^+^* strain NR2101, while those not shown (containing changes V230S, K231A, R232A, V233T, E237A, D238A, F239Y, and R240A) behaved like wild-type strain NR2101. Numbers represent the diameter (in millimeters) of the zone of inhibition or that of partial growth (parentheses) around a 6-mm-diameter disc containing an antibiotic. Download FIG S1, PDF file, 0.3 MB. Copyright © 2019 Simpson et al. 2019 Simpson et al. This content is distributed under the terms of the Creative Commons Attribution 4.0 International license.

## RESULTS

### The CTD of LptB is a unique domain essential for LPS transport.

The LptB ATPase contains domains that are conserved among NBDs of ABC transporters and that are essential for LPS transport and viability in E. coli (13, 15). Here, we discovered an essential, unique domain in LptB while working with a C-terminally tagged variant. Previous studies showed that C-terminally His-tagged LptB proteins hydrolyze ATP *in vitro* (15, 23–26). One of them, LptB-EHis_8_, when encoded by a gene borne in a plasmid, supports growth of E. coli cells lacking chromosomal *lptB* (15). However, we found that the resulting *lptB*-*EHis_8_* haploid cells are sensitive to hydrophobic antibiotics, a hallmark of LPS transport defects (see Fig. S1 in the supplemental material). Furthermore, we could not replace the native *lptB* gene with a chromosomal *lptB-His* allele. Instead, the only haploid strain that we isolated carried an altered chromosomal *lptB* allele (named *lptB1*) that we assume suppresses the lethality of the sought-after *lptB-His* mutant. This *lptB1* allele, which resulted from a frameshift mutation, encodes full-length LptB with a 34-residue C-terminal (CT) extension and is partially functional since haploid *lptB1* cells are sensitive to hydrophobic antibiotics (Fig. S1A) (27). We also found that the identity of the first amino acid of the CT extensions determines their impact on LptB function, explaining why *lptB1*, but not *lptB*-*His*, can substitute for chromosomal *lptB* (Fig. S1).

Since our results suggested a functional role for the CTD of LptB, we next characterized *in vivo* the function of LptB variants containing single substitutions in their CTD. We focused on the region encompassing residues 230 to 241 because it is fully conserved only in LptB orthologs and partially conserved in ATPases of closely related branched-chain amino acid transporters (Fig. S2; see also Text S1 in the supplemental material). We found that whereas substitutions at less-conserved positions did not affect function, those at the highly conserved residues Y234, L235, G236, and F239 abolished their ability to complement the loss of native *lptB* (Fig. 1B; see also Fig. S1E). These nonfunctional LptB variants were stable and exhibited dominant-negative effects, indicating they titrated wild-type LptB (LptB^WT^) and/or its partners LptFG into nonfunctional machinery (Fig. 1C; see also Data set S1 in the supplemental material). In fact, the dominance of the CTD mutant alleles was stronger than that of the catalytically dead *lptB*(*E163Q*) allele, suggesting that complexes containing LptB^WT^-LptB^E163Q^ heterodimers might still be somewhat functional whereas those containing LptB^WT^ and CTD-defective LptB variants might not. Together, these data demonstrate that the CTD of LptB is essential for LPS transport and suggest that the CTD might have a *trans* effect across monomers in the LptB dimer.

10.1128/mBio.01931-19.1 TEXT S1 Supplemental references for Fig. S2 and Data set S1. Download Text S1, PDF file, 0.1 MB. Copyright © 2019 Simpson et al. 2019 Simpson et al. This content is distributed under the terms of the Creative Commons Attribution 4.0 International license.

### The CTD of LptB forms critical interactions with the Walker A and switch helix domains.

**FIG 2:**
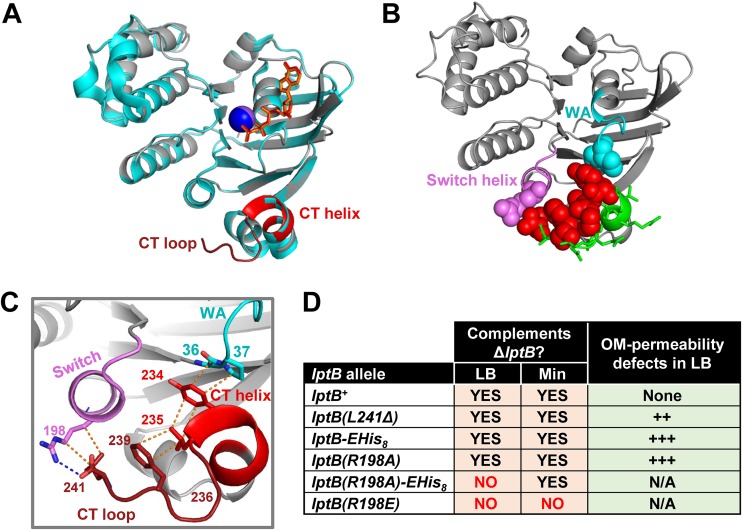
The CTD of LptB makes critical contact with the Walker A and switch helix. (A) Alignment (generated with align function in PyMol; root mean square [RMS] = 0.183) of ATP-bound structures of His_8_-LptB^E163Q^ (PDB 6MBN; gray) and LptB-EHis_8_^E163Q^ (PDB 4P33; cyan). The CTD of His_8_-LptB^E163Q^ is colored red. In PDB 6MBN, the Na^+^ atom is shown as a blue sphere and ATP as orange sticks. In 4P33, the Na^+^ atom is shown as a purple sphere and ATP as red sticks. (B) Cartoon representation of ATP-bound LptB structure (PDB 6MBN). CTD residues that, when substituted as in the Fig. 1 legend, resulted in no defects are shown as green sticks; those that caused total or partial loss of function are shown as red spheres. Functionally important CTD residues place the CTD helix and CTD loop such that they can interact with switch helix (violet) residue R198 (violet spheres) and Walker A (cyan) residues G36 and P37 (cyan spheres). (C) Detailed interactions of the functionally important CTD (red) residues shown in panel B. Dotted lines represent polar (blue) and nonpolar (orange) interactions. Y234 interacts with G36 and P37 in the Walker A motif (cyan); Y234, L235, and F239 stack together through hydrophobic interactions; G236 is likely critical for creating a turn that both facilitates stacking of L235 and F239 and properly orients L241; L241 interacts with R198 in the switch helix that follows the switch domain. (D) Functional characterization of mutant alleles encoding changes that disrupt the interaction between position R198 of the switch helix and the CTD loop. Table data indicate the ability of *lptB* alleles to complement a chromosomal Δ*lptB* allele in rich (LB) and minimal (Min) media. Haploid strains carrying alleles that complemented in LB were tested for increased OM permeability to antibiotics by disc diffusion assay. The relative increase in OM permeability is indicated with plus signs (+). Refer to Data set S1 for data and details.

Previously reported structures derived from CT-tagged LptB variants failed to fully resolve the CTD, suggesting that it might be flexible when tagged (15, 25, 26, 28). Therefore, to gain structural insight into the role of the wild-type CTD of LptB, we attempted to crystallize ADP- and ATP-bound E. coli LptB proteins with N-terminal His tags after confirming that they do not affect function. To trap the ATP-bound form, we used the catalytically dead LptB^E163Q^ protein as was done with CT-tagged LptB^E163Q^-EHis_8_ as previously described (15). We did not obtain a structure of ADP-bound His_8_-LptB, but we solved a 1.96-Å structure of ATP-bound His_8_-LptB^E163Q^. The new structure resembled that of LptB^E163Q^-EHis_8_, except that it included a fully resolved CTD (Fig. 2A; see also Fig. S3). The CTD is located at the LptB dimer interface below the ATP-binding sites, and it consists of a short helix (residues V230 to L235) followed by a turn and a loop (residues G236 to L241) (Fig. 2A). In ABC transporters, NBDs assemble in a head-to-tail conformation to form two ATP-binding sites, each composed of the Walker A and Walker B motifs of one monomer and the signature motif of the other monomer (18, 29). Within each LptB monomer, CTD-helix residue Y234 interacts with Walker-A invariant residue G36 and residue P37. In addition, CTD-helix residue L235 forms van der Waal interactions with CTD-loop residue F239 (Fig. 2B and C). Our *in vivo* data revealed that these interactions and placement of these residues are important for function, since altering them by substituting residues Y234, L235, G236, and F239 with alanine rendered LptB nonfunctional (Fig. 1; see also Fig. 2), while conservative substitutions (LptB^Y234F^ and LptB^F239Y^) that should have maintained their interactions had only a minor impact on function (Fig. S1E and F).

10.1128/mBio.01931-19.4 FIG S3 The CTD of LptB mediates intra- and intermonomer contacts through interactions with the switch helix. (A) Data collection and refinement statistics of PDB 6MBN structure of the ATP-bound His8-LptB^E163Q^ dimer. (B) Cartoon representation of the ATP-bound His8-LptBE163Q dimer (PDB 6MBN) showing that the carboxylic acid of the last residue in LptB, L241, forms ionic pairs (blue dotted lines) with residue R198 in the switch helix both within each monomer and across monomers. A bottom view of the LptB dimer is presented in which LptB monomers are colored cyan (monomer A) and light gray (monomer B), the switch helix containing R198 is colored pink, and the CTD is colored red. R198 and L241 are shown as sticks. (C) Detailed view of the interactions between residues R198 and L241. LptB monomers are colored cyan (monomer A) and light gray (monomer B). Download FIG S3, PDF file, 0.6 MB. Copyright © 2019 Simpson et al. 2019 Simpson et al. This content is distributed under the terms of the Creative Commons Attribution 4.0 International license.

The new ATP-bound structure also revealed a functional interaction between the carboxylate of CTD-loop residue L241, which is the last residue of LptB, and residue R198 from both monomers (Fig. 2B and C; see also Fig. S3). Residue R198 is located in the switch helix that follows the switch motif (or H loop) (18, 29). In LptB, the switch motif contains the essential histidine (H195) that interacts with the γ-phosphate of ATP and undergoes a large conformational change upon ATP hydrolysis to possibly allow the exit of P_i_ (15). Our previous results suggested that the R198-L241 interaction was functionally relevant, since haploid mutants producing LptB-R (in which the arginine added after residue L241 would likely cause a charge clash with R198) or CT-tagged LptB (in which the tag removes the carboxylate of L241 and likely interferes by steric hindrance) were partially defective in LPS transport (Fig. S1; see also Data set S1). To directly probe the relevance of this interaction, we constructed an LptB variant lacking L241 and found that haploid cells producing LptB^ΔL241^ were viable but were partially defective in LPS transport (Fig. S1E and F). Furthermore, we tested if alterations to R198 also resulted in LPS transport defects. Complementation and antibiotic-sensitivity analyses showed that *lptB*(*R198E*) was a total-loss-of-function allele and that *lptB*(*R198A*) was a partial-loss-of-function allele whose function could be further compromised by altering the CTD of LptB^R198A^ by adding a CT tag, as the *lptB*(*R198A*)-*EHis_8_* haploid strain was viable in minimal medium but not in LB (Fig. 2D; see also Data set S1). This conditional lethality reflects severely compromised LPS transport that supports growth under slow-growth conditions (i.e., minimal medium) but not under fast-growth conditions (i.e., LB) (30). Together, the data from our structure-function analyses suggest that the essential function of the CTD of LptB depends on the proper placement of the CTD helix (V230 to L235) and CTD loop (G236 to L241) through intramonomer interactions with G36 and P37 in the Walker A domain and with R198 in the switch helix. In addition, the interactions between L241 and R198 across dimers (Fig. S3) might be functionally relevant and responsible for the aforementioned strong dominance of nonfunctional CTD variants and their *trans* effect in LptB^WT^-LptB^CTD^ heterodimers (Fig. 1C).

### Changes in the ATP-binding domains suppress defects caused by alterations to the CTD of LptB.

**FIG 3:**
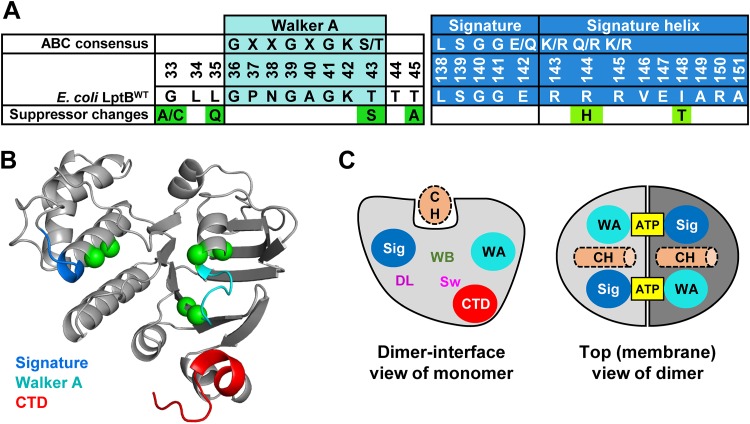
Changes to the ATP-binding sites suppress defects in the CTD of LptB. (A) Consensus sequences for the Walker A motif, signature motif, and signature helix of the ABC transporter family (PF00005) (56). Below each consensus sequence, the E. coli LptB^WT^ sequence and changes identified in suppressors are shown. (B) Cartoon representation of the structure of an His_8_-LptB^E163Q^ monomer showing the location of the suppressors of *lptB1* (represented as green spheres). The Walker A motif (cyan), signature helix (marine), and CTD (red) are colored in the structure. (C) The cartoon on the left shows the dimer interface view of an LptB monomer (light gray). The locations of the Walker A motif (WA; cyan) and signature (Sig; marine) motifs and of the CTD are marked. The locations of additional motifs important for function of ABC transporters are also shown as follows: “WB” marks the Walker B motif, “DL” the D loop, and “Sw” the switch loop. The Walker A and B motifs, together with the signature motif, constitute each ATP-binding site; the signature motif and D loop are thought to coordinate NBD-TMD coupling, and the switch loop is important for catalysis and possibly for release of P_i_ after hydrolysis. The structural groove on the top of each LptB monomer accommodates the coupling helix (CH, dotted light orange) of either LptF or LptG. The cartoon on the right shows the top view of the LptB dimer, where monomers appear in light and dark gray. Each ATP molecule is sandwiched between the Walker A motif and the signature motif of each monomer. The coupling helices (CH) are shown as dotted light orange cylinders.

To further understand the function of the CTD of LptB, we selected for suppressors of *lptB1*, which encodes a partially functional LptB variant with a 34-residue CT extension (Fig. S1). Suppressors selected on vancomycin or novobiocin plates carried intragenic mutations in *lptB1* and behaved in two distinct ways (previously described [27]): suppressors with substitutions G33A/C, L35Q, T43S, T45A, I148T, or S243Stop in LptB1 exhibited wild-type resistance to all antibiotics tested, while a suppressor with substitution R144H in LptB1 was resistant only to novobiocin (Fig. 3; see also Fig. S4). We do not further discuss the *lptB1*(*S243stop*) suppressor, which truncated the 34-residue CT extension to a single isoleucine, supporting our previous conclusion that the addition of an oligopeptide to the CTD negatively affects LptB function.

10.1128/mBio.01931-19.5 FIG S4 Structure-function analysis of *lptB1* suppressors. (A and B) OM permeability assay (A) and LptB immunoblots (B) of chromosomally encoded *lptB1* and suppressors performed as described in the Fig. S1 legend and in Materials and Methods. “*lptB^+^*” refers to strain NR754. (C) LptB immunoblot of samples from haploid strains producing LptB variants from pET23/42LptB derivatives performed as described in the Fig. S1 legend. (D) OM permeability assay of haploid cells carrying pET23/42LptB-EHis_8_ derivatives with the substitutions we found as suppressors of *lptB1*. Assays were performed as described in in the Fig. S1 legend. Data are representative of results from at least three independent experiments. Download FIG S4, PDF file, 0.2 MB. Copyright © 2019 Simpson et al. 2019 Simpson et al. This content is distributed under the terms of the Creative Commons Attribution 4.0 International license.

The remaining changes suppressing LptB1 localized to the Walker A and signature motif regions, which mainly constitute the two halves of each ATP-binding domain (Fig. 3; see also Fig. S5) (18). Changes localized in or flanking the Walker A motif (G33A/C, L35Q, T43S, T45A) also suppressed defects conferred by the *lptB-EHis_8_* allele (Fig. S4D). The remaining suppressors (I148T and R144H) mapped to the signature helix that follows the signature motif, the other half of the ATP-binding domain (Fig. 3) (31–35). The I148T substitution also suppressed *lptB1* and *lptB-EHis_8_* (Fig. S4). In contrast, as we reported earlier, the R144H substitution conferred resistance only to novobiocin, a known DNA gyrase inhibitor, because this antibiotic interacts with LptB at the LptFG interface to increase Lpt function (27). The fact that the combination of novobiocin and the R144H substitution suppressed *lptB1* and *lptB-EHis_8_* might suggest that the R144H change is a weak suppressor that needs the additional effect of novobiocin to fully suppress the CTD defect; however, as we describe below, this is not the case. Taking the results together, these suppressors revealed that the function of the CTD of LptB is related to ATP binding and/or hydrolysis.

10.1128/mBio.01931-19.6 FIG S5 Location of *lptB1* suppressors. (A and B) Cartoon representations of the structures of an His_8_-LptB^E163Q^ dimer (A) and monomer (B) showing the location of the suppressors of *lptB1* (represented as forest green spheres). The Walker A domain (cyan), signature motif (marine), and CTD (red) are colored in the X-ray structure. ATP is shown in yellow sticks. The location of additional motifs important for function of ABC transporters is also shown on the cartoons on the left. “WB” marks the Walker B motif, “DL” the D loop, “Sw” the switch loop, and “CH” a coupling helix of either LptF or LptG. Numbers in green shown in panel B represent the number of the residue altered in the suppressors. Download FIG S5, PDF file, 0.6 MB. Copyright © 2019 Simpson et al. 2019 Simpson et al. This content is distributed under the terms of the Creative Commons Attribution 4.0 International license.

### Defects caused by the R144H substitution and disruptions to the CTD loop-R198 interactions suppress one another.

**FIG 4:**
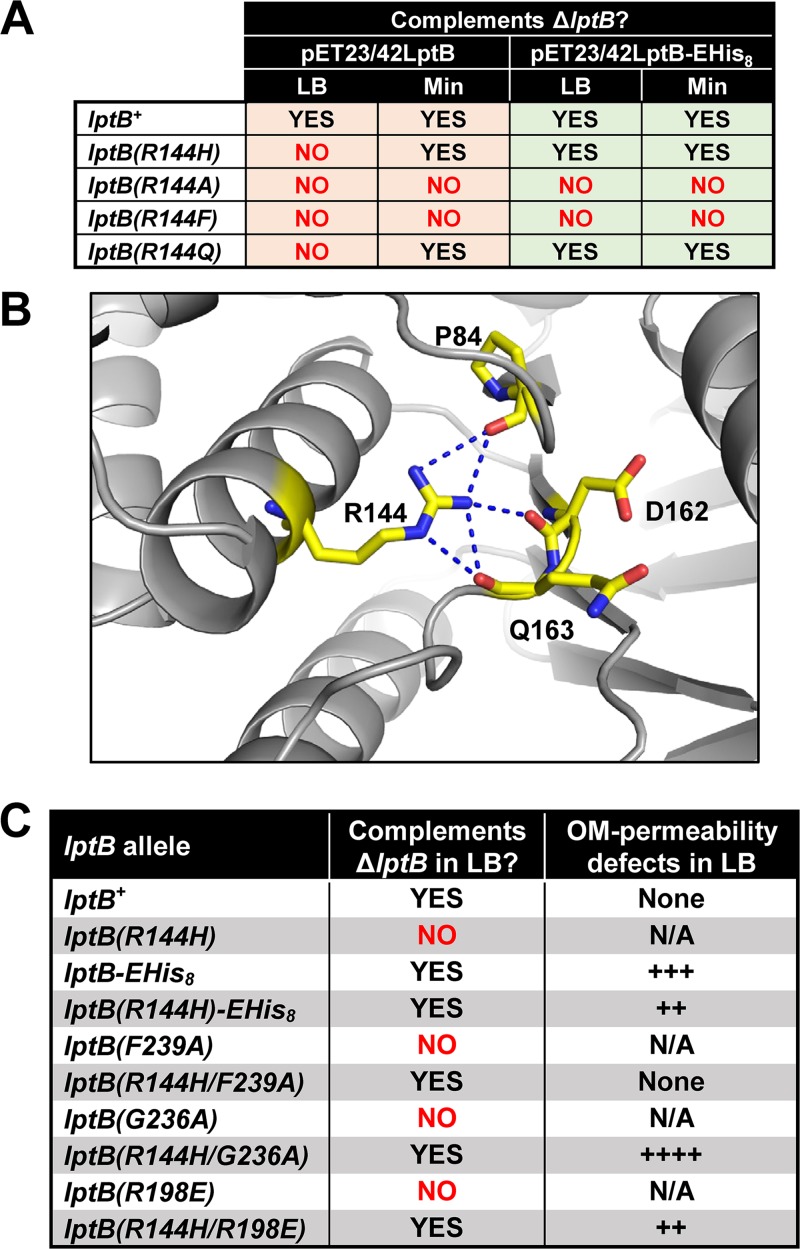
Specific changes to R144 and the CTD suppress each other. (A) Ability of variants with changes in R144 introduced in either LptB^WT^ or LptB-EHis_8_ to complement a Δ*lptB* allele in LB and in M63 minimal media supplemented with glucose (Min). (B) Interactions of R144 (marine) of ATP-bound His_8_-LptB_E163Q_ (PDB 6MBN) with the backbone of P84 of the Q-loop domain, D162, and Q163 in the Walker B domain (in wild-type LptB, E163 is the catalytic residue). (C) Cosuppression between the R144H and changes to R198 and the CTD loop. Mutant alleles were assayed for their ability to complement a chromosomal Δ*lptB* allele in LB. Haploid strains carrying alleles that resulted in complementation in LB were tested for increased OM permeability to antibiotics by disc diffusion assay. The relative levels of increases in OM permeability are indicated with plus signs (+). Refer to Data set S1 for complete data and details.

We next investigated the effect that the aforementioned suppressor mutations had in an otherwise wild-type *lptB* allele. Neither the introduction of I148T nor the changes in the Walker A region had an effect, except that some substitutions caused a reduction in LptB levels (Fig. S4). These substitutions also decreased LptB1 levels, further suggesting they suppress *lptB1* by conferring a functional change. When we introduced the R144H change into wild-type *lptB*, we generated a haploid *lptB*(*R144H*) strain that could grow in minimal medium but, surprisingly, not in LB (Fig. 4A). This conditional lethality is characteristic of severe Lpt defects (30), indicating that residue R144 is critical for LptB function. We considered that residue R144 might be functionally important because it forms polar interactions with the backbones of residues P84, D162, and Q163 (Fig. 4B), which are part of two motifs that are essential in ABC transporters. Residue P84 is in the Q loop, which is important for NBD-TMD communication; residues D162 and Q163 are in the Walker B motif, which is essential for ATP hydrolysis, with E163 being the catalytic glutamate in wild-type LptB (15, 18). Furthermore, the polar character is conserved at this position (R, K, or Q) in other ABC transporters (Fig. 3A). To investigate the relevance of these interactions, we substituted R144 with nonpolar alanine or phenylalanine, which abolished function, and with glutamine, which resulted in conditional lethality (Fig. 4A). These results suggested that polar contacts of R144 with the Walker B domain and/or the Q loop are critical for LptB function, implying that R144 might be important for ATP binding and/or hydrolysis and its coupling for the function of the TMDs.

The inability of haploid cells producing untagged LptB^R144H^ to grow in LB was surprising because those producing CT-altered LptB1^R144H^ are viable in this medium. This disparity in results suggested that the CTD extension in LptB1 fixes defects conferred by the R144H substitution. Nevertheless, this fix must not be complete, as OM permeability defects of *lptB1*(*R144H*) can be further suppressed by the addition of novobiocin (27). Since these data suggested that the R144H substitution and the CT extension in LptB1 suppressed each other, we tested if this cosuppression was specific to LptB1 or was applicable to other CTD alterations. We found that introducing different changes to the CTD of LptB^R144H^ also resulted in cosuppression (Fig. 4; see also Data set S1). Strikingly, whereas the *lptB*(*G236A*), *lptB*(*F239A*), and *lptB*(*R144H*) haploid strains were nonviable in LB, the *lptB*(*R144H/G236A*) and *lptB*(*R144H/F239A*) double mutant strains grew well in LB. In fact, the *lptB*(*R144H/F239A*) strain behaved similarly to the wild type. Thus, combining R144H, a severely compromising change in the signature helix, with F239A, a lethal change in the CTD, remarkably resulted in a double mutant that was phenotypically wild type.

We wondered whether the other suppressors of *lptB1* could also suppress the lethal mutations in the CTD loop. We found that Walker A substitution T45A, like R144H, could suppress the lethal effects of the F239A change but that the resulting *lptB*(*T45A/F239A*) double mutant was more sensitive to antibiotics than the wild-type-like *lptB*(*R144H/F239A*) mutant (Data set S1). Notwithstanding, the cosuppression between either an R144H or T45A substitution and alterations to the CTD loop (residues G236 to L241) was not applicable to defects in the CTD helix, as neither an R144H or T45A change could rescue the lethality of the *lptB*(*Y234A*) and *lptB*(*L235A*) alleles. Thus, changes to the ATP-binding sites specifically suppress alterations to the CTD loop but not to the CTD helix. This suggests that alterations to the CTD helix, in addition to misplacing the CTD loop, cause an additional lethal defect.

On the basis of the ATP-bound LptB structure (Fig. 2), we predicted that the G236A and F239A substitutions, as well as the addition of the His tag, would each result in misplacement of the CTD loop and thereby in disruption of its interaction with switch-helix residue R198. We therefore expected that the cosuppression between the R144H and CTD loop alterations was caused by the breaking of the CTD loop-R198 interaction and that changes to R198 would also suppress the effects of the R144H change. Indeed, the otherwise lethal R198E and conditionally lethal R144H substitutions suppressed each other (Fig. 4C; see also Data set S1). Thus, the lethal defects conferred by the R144H change were suppressed by interfering with the interaction between the CTD loop and residue R198, and vice versa. This strong cosuppression indicates that the function of signature-helix residue R144 is related to that of the interaction between the CTD loop and switch-helix residue R198. From the unexpected cosuppression of these substitutions, we infer that the individual changes affect the ATP cycle in opposite ways such that altering each function individually causes severe defects in LPS transport but these alterations compensate for each other when combined.

It is also worth noting that (i) the CT tags that we used were less defective than the lethal F239A change, (ii) F239A and R144H fully suppressed each other’s severe defects, and (iii) the cosuppression between the less-defective CT extensions in LptB1 or LptB-EHis_8_ and R144H was only partial and was capable of being further improved by novobiocin. Therefore, it appears as if novobiocin somehow further increases the levels of defects caused by the CT extensions of LptB1 or LptB-EHis_8_ such that their combined defects can better suppress those conferred by the R144H substitution. These observations also support the aforementioned proposal that the R144H and CTD substitutions affect the ATP cycle in opposite ways that compensate for each other when combined.

### A network of interactions connects the function of the signature helix and CTD of LptB.

**FIG 5:**
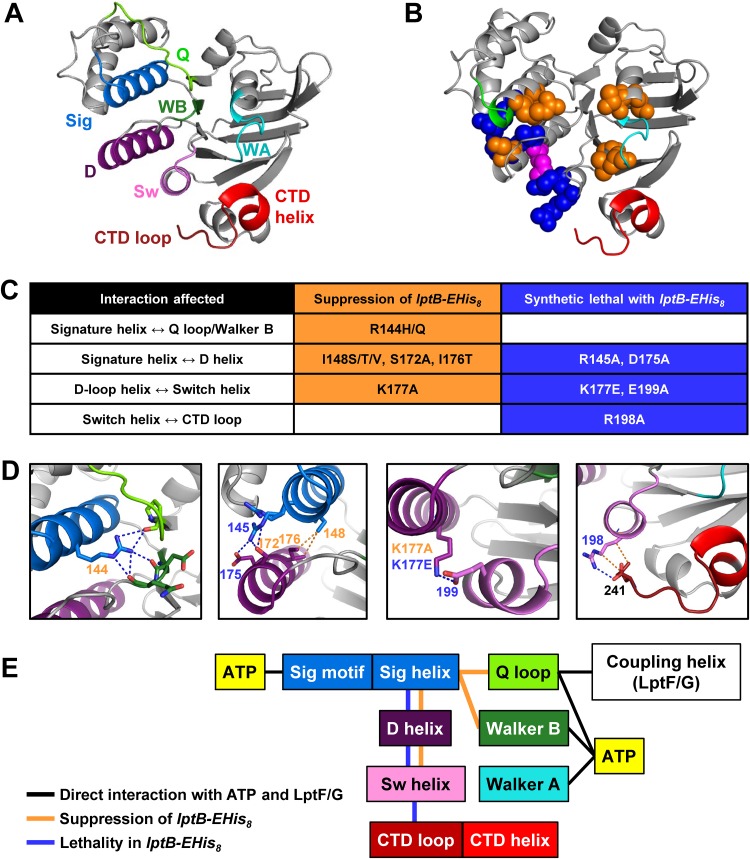
A network of helices connects the functions of the signature helix and the CTD of LptB. (A) Cartoon view of the structure of a monomer of ATP-bound His_8_-LptB^E163Q^ (PDB 6MBN). Relevant domains are the Q loop (Q; chartreuse), signature domain and helix (Sig; blue), Walker B domain (WB; forest green), Walker A domain (WA; cyan), D-loop helix (D; dark purple), switch helix (Sw; violet), CTD helix (red), and CTD loop (brick red). (B) Interactions observed in the ATP-bound crystal structure of His_8_-LptB^E163Q^ (dimer interface view of PDB 6MBN). Spheres correspond to residues that, when substituted, resulted in suppression (in orange) or synthetic lethality (in blue) or both depending on the substitution (pink). The Walker A and signature motifs are colored in cyan and green, respectively, and the CTD is shown in red. (C) Summary of substitutions that suppress (in orange) or cause lethality (in blue) when introduced into LptB-EHis_8_ (complete data are shown in Fig. 4; see also Fig. S6B and C). (D) Expanded views of structures of LptB (PDB 6MBN) with residues depicted as sticks and polar (blue) or nonpolar (orange) interactions depicted as dotted lines. Residue numbers are colored according to whether substitutions shown in panel C led to suppression (orange) or lethality (blue). (E) Schematics showing key domains and relevant physical and genetic interactions. Direct interactions between LptB domains and ATP or the coupling helices of LptF/G are marked with black lines. Orange lines represent interactions that, when altered, led to suppression of defects conferred by *lptB-EHis_8_*. Blue lines represent interactions that, when changed, led to synthetic lethality with *lptB-EHis_8_*.

Our genetic data revealed a functional link between the signature helix and the R198-CTD interaction. This connection would require a long-range network of interactions because these domains are not in close proximity (Fig. 5A). Upon examining the His_8_-LptB^E163Q^ structure, we predicted that the D-loop helix, which follows the D loop, mediates interactions between the signature helix (which contains R144) and the switch helix (which contains R198) (Fig. 5). The D loop is an essential motif in ABC transporters that is thought to coordinate NBD-TMD coupling and NBD dimerization (22, 36). We reasoned that if this network were relevant to coordinating the functions of the signature helix and the R198-CTD loop interaction, disrupting interactions along the network should then result in genetic interactions similar to those described for mutations that alter R144, R198, and the CTD loop. Indeed, this idea was supported by our earlier finding that an I148T change, which should alter one of the interactions along this putative network, suppressed the CTD-loop defective *lptB1* allele (Fig. 3; see also Fig. 5).

10.1128/mBio.01931-19.7 FIG S6 Synergistic effects of mutations combined with *lptB-EHis8* identifying a network of functionally related residues. (A) Sequence logo (generated as described in the Fig. 1D legend and Materials and Methods) of the conservation of residues in the signature, D-loop, and switch helices (locations of each are marked with thick lines). Below the logo, the sequence of E. coli LptB^WT^ is shown in black and a summary of the results of functional analysis of single amino acid substitutions designed to disrupt the observed interactions between R144 and the CTD is presented. Substitutions in pET23/42-LptB conferring no defects in strains carrying a chromosomal Δ*lptB* deletion are shown in green, those conferring partial loss of function are shown in orange, and those resulting in a total loss of function are shown in purple. (B) Ability of mutations that alter the indicated residue in either pET23/42LptB or pET23/42LptB-EHis_8_ to complement a Δ*lptB* allele in the indicated media as described in the Fig. S1A legend. (C) OM permeability of haploid strains carrying *lptB* mutations that exhibit effects synergistic with those of *lptB-EHis_8_*. Disc diffusion assays were performed on LB plates. Numbers indicate diameter (in millimeters) of the zone of total growth inhibition or that of partial growth (in parentheses). (D) LptB immunoblot of haploid *lptB-EHis_8_* strains carrying pET23/42-LptB-EHis_8_ grown in LB. (E) LptB immunoblot of samples from haploid *lptB* strains carrying pET23/42-LptB grown in minimal medium to allow growth of strains carrying conditionally lethal alleles. “WT” refers to NR2101, which produces LptB^WT^ from pET23/42-LptB. (F) LptB immunoblot of samples from merodiploid strains carrying the wild-type *lptB* allele and mutant *lptB* alleles carried in pET23/42-LptB grown in LB. “754” refers to haploid strain NR754, the wild-type strain with chromosomal *lptB^+^* and “WT” to NR2583, which produces LptB^WT^ from both the chromosome and pET23/42-LptB. (G) LptB immunoblot of samples from merodiploid strains carrying the chromosomal wild-type *lptB* allele and mutant *lptB-EHis_8_* alleles encoded in pET23/42-LptB-EHis_8_ grown in LB. “754” refers to haploid strain NR754; “WT” refers to NR1872, which produces LptB^WT^ from the chromosome and LptB-EHis_8_ from pET23/42-LptB-EHis_8_. Data are representative of results from at least three independent experiments. Download FIG S6, PDF file, 0.3 MB. Copyright © 2019 Simpson et al. 2019 Simpson et al. This content is distributed under the terms of the Creative Commons Attribution 4.0 International license.

To explore the relevance of this putative network, we introduced mutations expected to disrupt contacts between the signature, D-loop, and switch helices and tested their effects on wild-type and CT-loop-defective (*lptB-EHis_8_*) strains (Fig. 5; see also Fig. S6). We found that substitutions to networking residues in the signature (R144, R145, and I148), D-loop (D175 and K177) and switch (R198 and E199) helices caused LPS transport defects (Fig. S6). Moreover, these mutations either suppressed or were synthetically defective in *lptB-EHis_8_*, depending on the position or manner in which a position was altered (Fig. 5; see also Fig. S6). Thus, substitutions within the interaction network mimicked either the R144H change or those that break the R198-CTD interaction. Together, these data revealed a network of residues that functionally connects the signature helix and CTD loop of LptB and suggest that, depending on the manner in which the network is altered, LptB may favor one of two conformations or states.

### Decoupling of ATP hydrolysis and LPS extraction in complexes containing LptB^R144H/F239A^.

**FIG 6:**
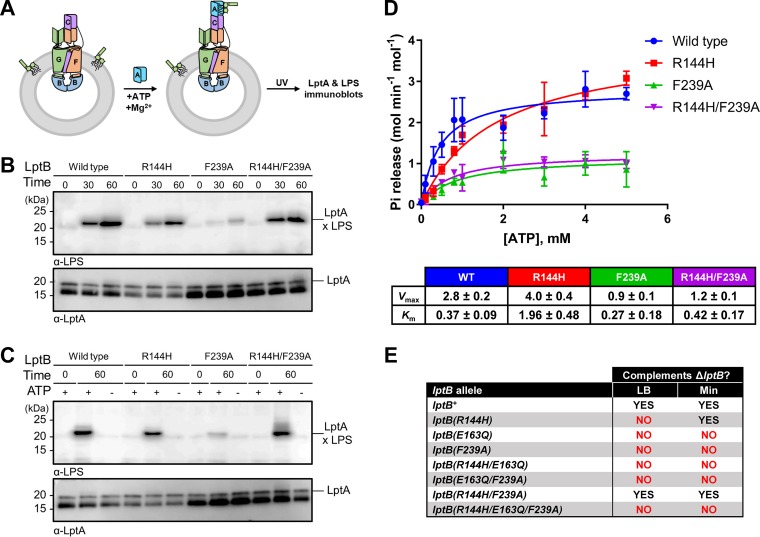
Decoupling of the ATPase activity of LptB and LPS extraction in mutant LptB_2_FGC complexes. (A) *In vitro* assay monitoring release of LPS from proteoliposomes containing LptB_2_FGC complexes to LptA^I36^*^p^*^Bpa^ by UV cross-linking and detection of LPS-LptA^I36^*^p^*^Bpa^ adducts by LPS immunoblotting. Refer to Materials and Methods for details. (B) Defects in the signature helix and CTD of LptB affect LPS release. LPS released from proteoliposomes containing LptB_2_FGC complexes with various LptB variants were UV-cross-linked to LptA^I36^*^p^*^Bpa^, and the resulting LPS-LptA^I36^*^p^*^Bpa^ adducts (marked LptA × LPS) were detected by LPS immunoblotting (top panel). Time (min) data indicate the incubation period of the LPS release assay in the presence of ATP. LptA immunoblots shown confirm total LptA levels (bottom panel). (C) The release of LPS shown in panel B was dependent on the presence of ATP. Time (min) data indicate the incubation period of the LPS release assay in the presence (+) or absence (−) of ATP. Immunoblots were performed as described for panel B. Data in panels B and C are representative of results from three independent biological replicates. (D) ATPase activity was monitored in the presence of various concentrations of ATP in the *in vitro* assay for LPS release from proteoliposomes containing LptB_2_FGC complexes with various LptB variants. Data represent averages and standard deviations of results determined using three different proteoliposome preparations for each LptB variant and a molybdate assay to monitor P_i_ release. (E) ATP hydrolysis is required for the cosuppression of defects conferred by the R144H and F239A substitutions in LptB, since introduction of the Walker B substitution E163Q into LptB^R144H/F239A^ rendered the otherwise fully functional protein unable to complement the loss of chromosomal *lptB*.

To further investigate the effect of altering the signature helix and CTD of LptB on LPS extraction and ATPase activity, we used an *in vitro* system with reconstituted LptB_2_FGC complexes (11). We prepared proteoliposomes containing LPS and different LptB_2_FGC complexes and compared the abilities of those complexes to transfer LPS to their soluble periplasmic partner LptA after extracting the glycolipid from the bilayer. For this, we used purified LptA^I36^*^p^*^Bpa^, which contains the UV-cross-linkable amino acid *para*-benzoyl phenylalanine (*p*BPA) at a position that interacts with LPS (10). If LPS were extracted and transferred to LptA^I36^*^p^*^Bpa^, exposure to UV would result in LPS-LptA adducts that could be detected using LPS immunoblotting (Fig. 6A). We found that, as previously reported (11), complexes containing LptB^WT^ transferred LPS to LptA in a time-dependent manner under conditions of saturating ATP concentrations (Fig. 6B). Compared to wild-type complexes, those containing LptB^R144H^ displayed some reduction in LPS extraction, while those with LptB^F239A^ were highly defective (Fig. 6B). In contrast, and as expected from their *in vivo* wild-type-like behavior, complexes containing LptB^R144H/F239A^ resembled wild-type complexes in their ability to extract LPS. For all complexes tested, LPS extraction required ATP (Fig. 6C). Furthermore, as previously reported (11), transfer of LPS to LptA required ATP hydrolysis, as shown by the lack of transport by complexes containing the catalytically dead LptB^E163Q^ (Fig. S7A).

10.1128/mBio.01931-19.8 FIG S7 ATP hydrolysis is required for LPS release from proteoliposomes. (A) Complexes containing the catalytically dead LptB^E163Q^ variant are defective in extracting LPS from liposomes. An assay monitoring LPS release from liposomes containing LptB_2_FGC complexes with LptB^WT^ and catalytically dead LptB^E163Q^ variants was performed. Released LPS was UV-crosslinked to LptA^I36^*^p^*^Bpa^, and the resulting LptA^I36^*^p^*^Bpa^ x LPS adducts were detected by LPS immunoblotting (top panel). Assays were initiated by the addition of ATP and incubation at 30°C for the indicated amount of time prior to crosslinking. LptA immunoblots are shown that confirm total LptA levels (bottom panel). Data are representative of results from three independent biological replicates. (B) *In vitro* assays for release of LPS from proteoliposomes containing LptB_2_CFG complexes with different LptB variants were performed in the presence of either ATP or nonhydrolyzable AMPPNP. After 60 min, LPS was cross-linked to LptA^I36^*^p^*^Bpa^ and the resulting LPS-LptA^I36^*^p^*^Bpa^ adducts (marked “LptA × LPS”) were detected by LPS immunoblot (top panel). Total levels of LptA^I36^*^p^*^Bpa^ in the assay were confirmed by LptA immunoblotting (bottom panel). We also did not detect LPS transport by complexes containing LptB^WT^ or LptB^R144H/F239A^ in using the nonhydrolyzable ATP analogs α,β-methylene ATP (ApCpp), 2-chloroadenosine β, γ-methylene ATP (AppCp), ATP-γS, and AppNH_2_. Download FIG S7, PDF file, 0.3 MB. Copyright © 2019 Simpson et al. 2019 Simpson et al. This content is distributed under the terms of the Creative Commons Attribution 4.0 International license.

Although the severe defect in LPS extraction displayed by complexes containing LptB^F239A^ correlated with its inability to complement *in vivo*, we were surprised that those containing LptB^R144H^ showed only a moderate defect, given that *lptB*(*R144H*) is a conditionally lethal allele. Since the aforementioned experiments were conducted under ATP-saturating conditions, we next investigated the ability of LptB_2_FGC complexes to bind and hydrolyze ATP in the presence of differing concentrations of ATP (Fig. 6D). Complexes containing LptB^R144H^ yielded *V*_max_ values similar to those yielded by wild-type complexes, but their *K_m_* values were higher, indicating that the binding affinity of LptB^R144H^ for ATP is reduced. Notably, unlike the levels seen with wild-type complexes, the *K_m_* of LptB^R144H^ complexes is above typical cellular ATP concentrations (37, 38). In contrast, the *K_m_* values of LptB^F239A^ and LptB^R144H/F239A^ complexes were similar to those of wild-type complexes, but their *V*_max_ values were reduced by approximately half, indicating a defect in catalysis.

Given that LptB^F239A^ and LptB^R144H/F239A^ complexes were similarly defective in ATP hydrolysis and that *lptB*(*F239A*) cells are nonviable whereas *lptB*(*R144H/F239A*) cells are wild-type-like, we tested whether the R144H substitution could bypass the need for LptB to hydrolyze ATP. To test this *in vivo*, we generated new mutant *lptB* alleles in which the E163Q change, which abolishes ATP hydrolysis (11, 15), was combined with R144H and R144H/F239A substitutions. None of these alleles complemented the loss of native *lptB* (Fig. 6E). Furthermore, *in vitro*, LptB^R144H/F239A^ complexes could not extract LPS from liposomes in the presence of various nonhydrolyzable ATP analogs (Fig. S7B). Therefore, in order to function, wild-type-like LptB^R144H/F239A^ hydrolyzes about half the amount of ATP as LptB^WT^.

Our genetic data demonstrate that the R144H and F239A substitutions have opposite, compensating effects on LptB function. The biochemical data show that the R144H substitution in the signature helix decreases the ability of LptB to bind ATP, while the F239A substitution in the CTD reduces its ability to hydrolyze ATP, but that combining the two substitutions suppresses defects in LPS transport by fixing the ATP binding defect without restoring the defect in ATP hydrolysis.

## DISCUSSION

We have found a pair of lethal mutations in *lptB* that individually impair two distinct steps of the ATP hydrolysis cycle but that together restore wild-type levels of LPS transport and cellular growth. Biochemical experiments showed that, individually, one of these changes (R144H) results in impaired ATP binding, as judged by an increase in *K_m_*, while the other (F239A) causes impaired turnover, as judged by a decrease in *V*_max_. Restoration of LPS transport in complexes containing both changes (LptB^R144H/F239A^) requires rescue only of ATP-binding activity (*K_m_*) and not of ATP hydrolysis activity (*V*_max_). The simplest explanation of these results is that LPS transport by wild-type LptB_2_FGC is normally driven by the defective step that becomes suppressed in LptB^R144H/F239A^ complexes, namely, ATP binding. If ATP hydrolysis determined LPS extraction, LptB^R144H/F239A^ complexes should still be defective in LPS transport because they remain defective in ATP hydrolysis. We therefore propose that ATP binding triggers the collapse of the LptFG cavity that moves LPS to the periplasmic bridge, while ATP hydrolysis is needed to return the LptB_2_FGC transporter to the ground state to initiate another LPS extraction.

**FIG 7:**
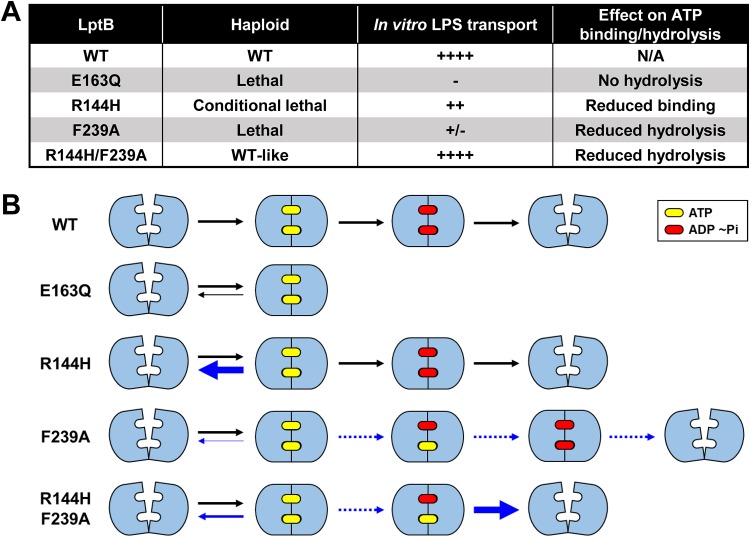
Model for the effects of the R144H and F239A changes on LptB dimerization. (A) Summary of relevant phenotypes to consider for the model shown in panel B. In the absence of ATP, the wild-type LptB (WT) dimers are in the open-dimer state. Upon binding to ATP, the LptB dimer closes; ATP hydrolysis at both ATP-binding sites is then needed to open the dimer such that the transporter returns to its ground state and initiates another transport cycle. The LptB^E163Q^ variant can bind ATP, but it cannot hydrolyze it. We propose that a complex containing LptB^E163Q^ can undergo only one round of transport and stalls in a closed-dimer state. The LptB^R144H^ variant has lower affinity for ATP, disfavoring the closed-dimer state, and therefore shows an increased tendency to reopen (indicated by reverse blue arrow); consequently, transporters with LptB^R144H^ do not function efficiently, and haploid *lptB*(*R144H*) mutants are viable only under conditions of slow growth. In contrast, LptB^F239A^ has a defect that favors the closed-dimer state (thinner blue reverse arrow) and decreases the rate of the subsequent ATP hydrolysis reactions such that hydrolysis occurs at only one site during a time span that would normally allow it to occur at both sites. Because hydrolysis of both ATP molecules is required to open the LptB dimer and because LptB^F239A^ favors the closed-dimer state (dotted blue lines), the resetting of the transporter is highly defective; consequently, complexes with LptB^F239A^ are defective in both ATP hydrolysis and LPS transport. In the LptB^R144H/F239A^ complexes, the opposite effects of the R144H and F239A substitutions on the stability of the nucleotide-bound states compensate for each other. As a result, the instability of the ATP-bound closed-dimer state caused by R144H is somewhat lowered by the higher stability of the closed-dimer state induced by the F239A change (thinner blue arrow); in addition, the miscoordination of the hydrolysis activities at the two ATP-binding sites and the resulting difficulty in opening an ATP-ADP dimer caused by the F239A substitution are compensated for by the lower stability of the closed-dimer state caused by the R144H change. Thus, the R144H/F239A combination allows the complexes to reset to the open-dimer state without fixing the defect in the ATP hydrolysis rate such that the double mutant complexes hydrolyze less ATP when transporting LPS.

The restoration of LPS transport in LptB^R144H/F239A^ must be driven by the addition of opposing defects caused by each change. LptB^R144H^ has impaired ATP binding such that it is easier to open the ATP-bound dimer (see bold blue arrow in Fig. 7B). It follows that the F239A change can fix (suppress) the defect conferred by the R144H change because it stabilizes the conformation in which the LptB dimer is closed around ATP (see thin blue arrow in Fig. 7B). This conclusion is supported by the kinetic characterization of LptB^F239A^. Moreover, the fact that the low *V*_max_ of LptB^F239A^ was not suppressed by the R144H change implies that this catalytic defect (see dotted blue arrows in Fig. 7B) cannot be caused simply by the increased stabilization of the ATP-bound state; instead, LptB^F239A^ must have an additional yet-to-be determined defect. Nevertheless, the lowered dimer stability conferred by the R144H change allows LptB^R144H/F239A^ to reopen after hydrolysis of a single ATP molecule (see bold blue arrow for double mutant in Fig. 7B). It is formally possible that the double LptB^R144H/F239A^ mutant complex eliminated the requirement of ATP hydrolysis for LPS transport; however, all attempts to observe transport with nonhydrolyzable ATP analogues or in the presence of the catalytically dead E163Q change were unsuccessful.

Further, upon being tested for dominance in merodiploid strains, production of LptB^F239A^ (or other total-loss-of-function CTD-defective variants) in the presence of wild-type LptB resulted in greater defects in LPS transport than coproduction of the catalytically dead LptB^E163Q^ and wild-type LptB. This difference implies that CTD defects cause a worse defect than simply that of killing catalytic activity. The simplest explanation is that, while LptB^E163Q^ cannot hydrolyze ATP, mixed LptB^WT^-LptB^E163Q^ complexes can turn over (i.e., open the dimer) at a higher rate than LptB^WT^-LptB^F239A^ complexes. This difference in the levels of dominance of the defective variants illustrates that LptB_2_FGC can, in certain circumstances, turn over with defective ATP hydrolysis and that LptB^F239A^ cannot be defective only in ATP hydrolysis. Indeed, these results are consistent with the idea that the F239A change results in an overly stable dimer that is defective in turnover, even in mixed LptB^WT^-LptB^F239A^ complexes.

Our studies have identified interdomain interactions that functionally connect the distant signature helix and the CTD in LptB. Key features identified to be important here are conserved in other ABC transporters. For example, residue Q140 of MalK, the NBD of the MalK_2_FG maltose importer, is equivalent to R144 in LptB, and substitutions at Q140 in MalK result in nonfunctional transporters owing to the presence of dimers that are trapped in either an open or a closed conformation (39, 40). In addition, we found that the D-loop helix is central in the network that functionally links the signature helix containing R144 and the CTD loop containing F239 in LptB. In the TAP (transporter associated with antigen processing) ABC transporter, a change in the D loop was shown to decrease affinity of the NBD dimer (36), while its D-loop helix was also demonstrated to play a critical role in the dimerization of the NBDs (41). Additional studies will be needed to better understand how the network of functional interactions that we have uncovered couples the movement of LptFG with the dimerization state of LptB, as well as how conserved their role is across ABC transporters.

## MATERIALS AND METHODS

### Strains and growth conditions.

All strains are listed in Data set S1 in the supplemental material and were derived, unless otherwise indicated, from NR754, an *ara^+^* derivative of MC4100 (42, 43). Cells were grown with aeration at 37°C in LB or M63 minimal media with 0.2% (wt/vol) glucose. Plates were prepared with 1.5% (wt/vol) agar, and top agar was prepared with 0.75% (wt/vol) agar. When applicable, the medium was prepared with the addition of ampicillin (125 μg/ml), isopropyl-β-d-1-thiogalactopyranoside (IPTG; 0.16 mM), kanamycin (30 μg/ml), novobiocin (5 or 33 μg/ml), tetracycline (25 μg/ml), vancomycin (75 μg/ml), or X-Gal (5-bromo-4-chloro-3-indolyl-β-d-galactopyranoside; 33 μg/ml).

### Mutant allele construction.

Mutant alleles of *lptB* were constructed in pET23/42LptB by site-directed mutagenesis (SDM) PCR and confirmed by DNA sequencing with the primer T7 seq (15). SDM PCR was performed with PfuTurbo DNA polymerase and primers designed per the protocol specified for QuikChange (Agilent Technologies, Inc., Santa Clara, CA). All primers are listed in Data set S1. PCR products were then digested with DpnI (New England Biolabs, Ipswich, MA) and electroporated into DH5α competent cells.

### Functional analysis of mutant alleles.

Functionality of *lpt* alleles was assessed as previously described (44). Briefly, pET23/42LptB-carried mutant alleles were transformed into strain NR2050 carrying a chromosomal *lptB* deletion and a wild-type copy of *lptB* on segregation-defective plasmid pRC7 (15, 45). Transformants were selected on LB containing ampicillin, IPTG, and X-Gal. Without selective pressure, the pRC7-derived plasmid is easily lost, and that loss is monitored by detection of the presence of an encoded *lacZ* gene (45). Mutant alleles that could sustain cell viability lost the pRC7-derived plasmid in the resulting strain and had white colonies (LacZ^−^). In contrast, loss-of-function alleles yielded only blue colonies (LacZ^+^) because the strain required the pRC7-carried copy of *lptB*. Functionality was checked on LB and glucose minimal solid media. Mutant alleles that were unable to complement deletion of chromosomal *lptB* on minimal medium were transformed into NR754 to generate *lptB* merodiploid strains and to assess dominance.

### OM permeability assays.

Sensitivity to hydrophobic antibiotics (bacitracin, novobiocin, erythromycin, and rifampin) was used to assess OM permeability of strains that were haploid or merodiploid with respect to *lptB* with pET2342LptB-encoded mutant alleles. Disc diffusion assays were performed as described previously (15).

### Immunoblotting of LptB variants.

Strains were grown overnight in glucose minimal medium or LB broth as indicated, and samples were normalized for differences in cell density. The resulting samples were subjected to electrophoresis on 12% SDS polyacrylamide gels. Proteins were transferred to polyvinylidene difluoride (PVDF) membranes (Roche Diagnostics, Basel, Switzerland) and probed with anti-LptB antisera (1:10,000 dilution) followed by anti-rabbit horseradish peroxidase (HRP) antibodies (GE Amersham, Chicago, IL) (1:10,000 dilution). Clarity Western ECL substrate (Bio-Rad, Hercules, CA) was used to develop signal and a ChemiDoc XRS+ system, and ImageLab 5.2.1 software (Bio-Rad) was used for imaging.

### Suppressor analysis of *lptB1*.

Several cultures of NR1768, which contains the *lptB1* allele, were grown overnight in LB as previously described (27). From each culture, a 100-μl volume was plated onto LB agar containing either novobiocin (33 μg/ml) or vancomycin (75 μg/ml). A kanamycin resistance cassette immediately downstream of *lptB1* was used to test for linkage to *lptB1* by P1vir transduction. When the suppressor phenotype was 98% to 100% linked to the kanamycin resistance cassette, this suggested the suppressor mutations were located in *lptB1*. PCR was performed to amplify the chromosomal locus with primers 5LptB77up and 3LptB50down. The resulting PCR products were sequenced. Each of the suppressors described here had a single-base-pair missense mutation in *lptB*, including the following: a G98C mutation resulting in a G33A substitution, a G97T mutation resulting in a G33C substitution, a T104A mutation resulting in an L35Q substitution, a C128G mutation resulting in a T43S substitution, a A133G mutation resulting in a T45A substitution, a G431A mutation resulting in an R144H substitution, a T443C mutation resulting in an I148T substitution, and a C728A mutation resulting in a stop codon after codon 242.

### Construction of pET28b(+)-His8TEV-LptB(E163Q).

pCDFduet-His6LptB(E163Q)-LptFG (15) was modified to lengthen the His tag and to include a tobacco etch virus (TEV) protease cleavage site by use of the primers His8TEV-LptB-fwd and His8TEV-LptB-rev (Data set S1). Restriction enzymes NcoI and EcoRI were used to transfer the gene encoding His_8_TEV-LptB(E163Q) into pET28b+.

### Expression, purification, and crystallization of His-LptB(E163Q).

Overnight cultures of KRX E. coli containing pET28b(+)-His_8_TEV-LptB(E163Q) were diluted 1:100 into fresh LB (Miller) with 50 μg/ml kanamycin, grown at 37°C to an optical density at 600 nm (OD_600_) of approximately 1.0, and then cooled to 16°C, at which point expression was induced by addition of 0.05% (wt/vol) l-rhamnose monohydrate (Sigma-Aldrich, St. Louis, MO) and 100 μM IPTG. After 18 h of expression, cells were collected by centrifugation at 4,200 × *g* and 4°C for 20 min. Cells from a 1.5-liter culture were resuspended in 40 ml buffer A (150 mM NaCl, 20 mM Tris [pH 8], 20% [vol/vol] glycerol, 0.5 mM dithiothreitol [DTT]; Sigma-Aldrich), supplemented with 20 mM imidazole, 0.5% (wt/vol) n-dodecyl β-d-maltoside (DDM; Anatrace, Maumee, OH), 2 mM ATP (Sigma-Aldrich), 1 mM phenylmethylsulfonyl fluoride (PMSF; Sigma-Aldrich), 100 μg/ml lysozyme (Sigma-Aldrich), and 50 μg/ml DNase I (bovine; Sigma-Aldrich). Cells were lysed by 3 passages through an Emulsiflex C3 homogenizer (Avestin, Ottawa, CA) at 15,000 lb/in^2^. Unbroken cells were removed from the lysate by centrifugation at 12,000 × *g* and 4°C for 10 min, and then other insoluble debris was removed by ultracentrifugation at 100,000 × *g* and 4°C for 30 min. Nickel-nitrilotriacetic acid (Ni-NTA) affinity resin was preequilibrated with buffer A and 20 mM imidazole, and the clarified supernatant was rocked with the resin for 1 h at 4°C. After incubation and flowthrough of the clarified lysate, the resin was washed with 20 column volumes (cv) buffer A with 20 mM imidazole and then 15 cv buffer A with 40 mM imidazole. Protein was eluted with 3 cv buffer A with 200 mM imidazole and the eluate supplemented with 2 mM ATP and 1 mM EDTA (Sigma-Aldrich). The protein sample was concentrated using an Amicon 10-kDa-molecular-weight-cutoff centrifugation filter (EMD Millipore, Burlington, MA) and further purified by gel filtration using a Superdex 200 10/300 GL column (GE Healthcare, Chicago, Illinois) with a mixture consisting of 150 mM NaCl, 20 mM Tris (pH 8), 20% (vol/vol) glycerol, and 0.5 mM Tris(3-hydroxypropyl)phosphine (THP; EMD Millipore). Peak fractions containing purified protein were pooled, supplemented with 2 mM ATP and 1 mM EDTA, concentrated to 20 mg/ml, and then either flash frozen and stored at –80°C or diluted 1:1 with buffer containing 150 mM NaCl, 20 mM Tris (pH 8), 0.5 mM THP, 2 mM ATP, and 1 mM EDTA. The diluted protein samples (∼10 mg/ml) were used to set crystallization trials using previously published conditions (15). The best diffracting crystals appeared in hanging drops set with a 1:1 protein-to-reservoir ratio, with the reservoir containing 28% polyethylene glycol (PEG) 4000 (Sigma-Aldrich), 100 mM MES (morpholineethanesulfonic acid; Tocris Biosciences, Bristol, United Kingdom) (pH 6.75), and 600 mM NaCl. After 3 weeks of growth, crystals were harvested, cryoprotected with a mixture containing 28% PEG 4000, 100 mM MES (pH 6.75), 600 mM NaCl, 20% glycerol, 2 mM ATP, and 1 mM EDTA, and then flash-frozen in liquid nitrogen.

### Data collection, data processing, and structure refinement.

X-ray diffraction data were collected at 0.97918 Å on beamline 24-ID-E at Argonne National Laboratory. The His_8_-LptB^E163Q^ crystals belonged to space group C222_1_. Data were indexed and integrated in XDS (46) or iMosflm (47) and were then scaled in the CCP4 suite (48) program AIMLESS (49). Molecular replacement was performed using Phaser (50) with the ATP-bound LptB^E163Q^-EHis_8_ structure (PDB 4P33) (15). Manual building was performed using Coot (51), while automated refinement was performed in Phenix (52). Drops of His_8_-LptB^E163Q^ were crystallized in the presence of ATP, and the structure of the inactive variant contained high electron density corresponding to ATP. This is consistent with previous studies of LptB (15, 27). All software was accessed through the SBGrid consortium (53). Data collection and refinement statistics are given in Fig. S3A in the supplemental material.

### Purification of LptB_2_FGC complexes.

LptB_2_FGC complexes containing different LptB variants were purified as previously described, with slight modifications (54). Overnight cultures of BL21(λDE3) E. coli containing pCDFduet-LptB-LptFG (wild-type or mutant LptB, as applicable) and pET22/42-LptC-His_7_ were diluted 1:100 into LB containing carbenicillin and spectinomycin. Cells were grown at 37°C to an OD_600_ of ∼0.8 and were then cooled to 18°C. IPTG (200 μM) was added after 20 min, and cells were grown overnight. Cells were harvested by centrifugation (4,200 × *g*, 20 min), resuspended in lysis buffer (50 mM Tris [pH 7.4], 300 mM NaCl, 1 mM PMSF, 100 μg/ml lysozyme, 50 μg/ml DNase I), homogenized, and subjected to passage through an EmulsiFlex-C3 high-pressure cell disruptor 3 times. The cell lysate was centrifuged (10,000 × *g*, 10 min), and the supernatant was further centrifuged (100,000 × *g*, 45 min). The resulting pellets were resuspended and solubilized in solubilization buffer (20 mM Tris [pH 7.4], 300 mM NaCl, 10% glycerol, 5 mM MgCl_2_, 1% [wt/vol] DDM [Anatrace Maumee, OH], 50 μM PMSF, 15 mM imidazole, 2 mM ATP) and rocked at 4°C for 1 h. The mixture was centrifuged (100,000 × *g*, 30 min) and the supernatant was rocked with Ni-NTA Superflow resin (Qiagen) for 1 h. The resin was then washed with affinity buffer (20 mM Tris [pH 7.4], 300 mM NaCl, 10% glycerol, 0.01% DDM, 0.01% LMNG [lauryl maltose neopentyl glycol; Anatrace]) containing 35 mM imidazole. Protein was eluted with affinity buffer containing 200 mM imidazole, concentrated using a 100-kDa-molecular-weight-cutoff Amicon Ultra centrifugal filter (Millipore), and purified by size exclusion chromatography on a Superdex 200 increase column in buffer containing 20 mM Tris-HCl (pH 7.4), 300 mM NaCl, 0.005% (wt/vol) LMNG, and 10% glycerol. The His tag was cleaved by overnight incubation with restriction-grade thrombin (Sigma). The solution was spiked with 8 mM imidazole, and the uncleaved protein was removed by passage through Ni-NTA resin and benzamidine Sepharose. Purified protein was either used within 24 h or flash frozen and stored at –80°C.

### Purification of LptA^I36^*^p^*^BPA^.

LptA^I36^*^p^*^BPA^ was purified as previously described, with slight modifications (10). Briefly, Bl21 (λDE3) E. coli cells containing pSup-BpaRS-6TRN and pET22b-LptA(I36Am) were grown to an OD_600_ of ∼0.6 at 37°C in LB media containing 50 μg/ml carbenicillin, 30 μg/ml chloramphenicol, and 0.8 mM *p*BPA (BaChem, Bubendorf, Switzerland). Cells were then induced with 50 μM IPTG; allowed to grow for 2 h; harvested; resuspended in a mixture containing 50 mM Tris-HCl (pH 7.4), 250 mM sucrose, and 3 mM EDTA; incubated on ice for 30 min; and pelleted (6,000 × *g*, 10 min). The supernatant was supplemented with 1 mM PMSF and 10 mM imidazole and pelleted (100,000 × *g*, 30 min). The supernatant was incubated with Ni-NTA resin, which was then washed twice (20 column volumes of 20 mM Tris-HCl [pH 8.0], 150 mM NaCl, 10% [vol/vol] glycerol, and 20 mM imidazole). LptA was eluted twice (2.5 column volumes of wash buffer supplemented with an additional 180 mM imidazole), concentrated using a 10-Da-cutoff Amicon centrifugal concentrator (Millipore), flash frozen, and stored at –80°C until use.

### Preparation of LptB_2_FGC liposomes.

Liposomes were prepared using a detergent dilution method as previously described (11). Aqueous E. coli polar lipid extract (Avanti Polar Lipids Inc.) (30 mg/ml) and aqueous LPS from E. coli EH100 (Ra mutant; Sigma) (2 mg/ml) were sonicated briefly for homogenization. A mixture of 20 mM Tris-HCl (pH 8.0), 150 mM NaCl, 7.5 mg/ml E. coli polar lipids, 0.5 mg/ml LPS, and 0.25% DDM was prepared and kept on ice for 10 min. Purified LptB_2_FGC was added to a final concentration of 0.86 μM, and the mixture was left on ice for 20 min. The mixture was diluted 100-fold with cold 20 mM Tris-HCl (pH 8.0) and 150 mM NaCl and kept on ice for 20 min. The proteoliposomes were pelleted (300,000 × *g*, 2 h, 4°C), resuspended in 20 mM Tris-HCl (pH 8.0) and 150 mM NaCl, diluted 100×, and centrifuged (300,000 × *g*, 2 h, 4°C). The pellets were resuspended in a mixture of 20 mM Tris-HCl (pH 8.0), 150 mM NaCl, and 10% glycerol (250 μl per 100 μl of the original predilution solution), homogenized by sonication, flash frozen, and stored at –80°C until use.

### LPS release assay.

The levels of release of LPS from proteoliposomes to LptA were measured as previously described, with slight modifications (11). Assays used 60% proteoliposomes (by volume) in a solution containing 50 mM Tris-HCl (pH 8.0), 500 mM NaCl, 10% glycerol, and 2 μM LptA^I36^*^p^*^BPA^. Reactions were initiated by the addition of ATP (or nonhydrolyzable ATP analogues ATP and AMP-PNP [adenylyl-imidodiphosphate] from Sigma, St. Louis, MO; all other analogues were from Jenna Bioscience, Jena, Germany) and MgCl_2_ (final concentrations of 5 mM and 2 mM, respectively) and proceeded at 30°C. Aliquots (33 μl) were removed from the reaction mixtures and irradiated with UV light (365 nm) on ice for 10 min using a B-100AP lamp (Fisher Scientific, Hampton, NH). Following UV irradiation, samples were added to a mixture consisting of 297 μl cold 20 mM Tris-HCl (pH 8.0), 150 mM NaCl, and 0.2% DDM. To precipitate the protein, 330 μl 20% trichloroacetic acid (Sigma) was added, after which the precipitated protein was collected by centrifugation. The precipitates were resuspended in 50 μl 1:1 2× SDS-PAGE sample loading buffer supplemented with 2% β-mercaptoethanol–1 M Tris-HCl (pH 8.0) and boiled for 10 min, and proteins were separated using Tris-HCl 4% to 20% polyacrylamide gradient gels with Tris-glycine running buffer. Proteins were transferred onto Immun-Blot PVDF membranes (Bio-Rad). Mouse monoclonal antiserum against the LPS core (Hycult Biotechnology, Uden, The Netherlands), rabbit anti-LptA antisera (11), sheep anti-mouse horseradish peroxidase (HRP) conjugate secondary antibody (GE Amersham), and donkey anti-rabbit HRP conjugate secondary antibody (GE Amersham) were used for the immunoblots. Bands were visualized using ECL Prime Western blotting detection reagent (GE Amersham) and an Azure c400 imaging system.

### ATPase assay.

ATPase assays were done using a modified molybdate method as previously reported (11). Assays used 60% proteoliposomes (by volume) in a mixture containing 50 mM Tris-HCl (pH 8.0), 500 mM NaCl, and 10% glycerol. Reactions were initiated by the addition of ATP and MgCl_2_ (final concentrations of 5 mM and 2 mM, respectively, unless otherwise indicated) and run at 30°C. Aliquots (25 μl) were taken at 0, 20, 40, and 60 min. Reactions were quenched with an equal volume of 12% sodium dodecyl sulfate (SDS). The amounts of P_i_ were determined using a colorimetric method, and potassium phosphate was used as a standard (55). Reagents were obtained from Sigma-Aldrich. After the addition of SDS, a mixture containing 50 μl of 30 mg/ml ascorbic acid, 0.5 N HCl, 5 mg/ml ammonium molybdate, and 6% SDS was added. The samples were incubated at room temperature for 7 min, and 75 μl of an aqueous solution containing 20 mg/ml sodium citrate tribasic dihydrate, 2 mg/ml sodium arsenite, and 2% (vol/vol) acetic acid was added. The absorbance at 850 nm was measured using a Spectramax Plus 384 plate reader (Molecular Devices, CA) after 20 min. Error bars indicate the standard deviations of the average rates measured over 3 biological replicates.

### ATPase assays for measuring *V*_max_ and *K_m_*.

Assays were performed as described above for the ATPase assays done under LPS release conditions with the following modifications. Assays included 30% proteoliposomes (by volume) in a mixture containing 50 mM Tris-HCl (pH 8.0), 500 mM NaCl, and 10% glycerol and were initiated by the addition of ATP and MgCl_2_ (the final concentration of ATP was adjusted as indicated; 2 mM final concentration of MgCl_2_) and run at 33°C. Aliquots (5 μl) were taken at 0, 30, 60, and 90 min. They were quenched with an equal volume of 12% sodium dodecyl sulfate (SDS). The amounts of P_i_ were determined using a published colorimetric method, and potassium phosphate was used as a standard as described above and previously (55). Error bars indicated the standard deviations of the rates measured over 3 biological replicates.

### Data availability.

The atomic coordinates and structure factors have been deposited at the RCSB Protein Data Bank with accession number 6MBN.
