# Supplementary material for: Combining Mutations That Inhibit Two Distinct Steps of the ATP Hydrolysis Cycle Restores Wild-Type Function in the Lipopolysaccharide Transporter and Shows that ATP Binding Triggers Transport
Source: mBio. 2019 Aug 20;10(4):e01931-19. doi: 10.1128/mBio.01931-19 (PMC6703430; doi:10.1128/mBio.01931-19)
Supplement: DATA SET S1 [file mBio.01931-19-sd001.pdf]

DATASET 1

Combining mutations that inhibit two distinct steps of the ATP hydrolysis cycle restores wild-type function in the lipopolysaccharide transporter and shows that ATP binding triggers transport

Brent W. Simpson, Karanbir S. Pahil, Tristan W. Owens, Emily A. Lundstedt, Rebecca M. Davis, Daniel Kahne, and Natividad Ruiz

Loss-of-function substitutions at the CTD of LptB confer dominant-negative phenotypes.

		Zone of inhibition[a]			
***lptB* alleles (chromosomal/plasmid)**	**Altered domain**	**Bacitracin**	**Novobiocin**	**Erythromycin**	**Rifampin**
***lptB*$^+$ (haploid)**	None	(10)	(10)	(13)	9 (10)
***lptB*$^+$/*lptB*$^+$**	None	(8)	(10)	(14)	10
lptB*$^+$/*lptB(E163Q)	Walker B	(10)	8 (9)	11 (15)	13
lptB*$^+$/*lptB(Y234A)	CTD helix	12 (15)	14	14	20
lptB*$^+$/*lptB(L235A)	CTD helix	14 (16)	15	15	21
lptB*$^+$/*lptB(G236A)	CTD loop	10	13	11 (15)	17
lptB*$^+$/*lptB(F239A)	CTD loop	11	16	11 (15)	16

[a]OM permeability of merodiploid strains carrying native chromosomal *lptB* and various *lptB* alleles in pET23/42 was assessed by disc diffusion assays in LB. Numbers indicate diameter (in mm) of the zone of total growth inhibition or that of partial growth (in parenthesis). Data are representative of at least three independent experiments.

OM-permeability defects of haploid strains with mutations in *lptB* that disrupt the interaction between the switch helix and CTD loop.

	Zone of inhibition[a]			
***lptB* allele**	**Bacitracin**	**Novobiocin**	**Erythromycin**	**Rifampin**
***lptB*⁺**	(8)	(8)	8 (10)	9 (10)
lptB(ΔL241)	11	14 (21)	13 (16)	12
lptB-EHis$_8$	13	14 (22)	16 (20)	13
lptB(R198A)	15	15 (24)	15 (20)	13

[a]OM permeability of haploid strains carrying various *lptB* alleles in pET23/42 was assessed by disc diffusion assays in LB. Numbers indicate diameter (in mm) of the zone of total growth inhibition or that of partial growth (in parenthesis). Data are representative of at least three independent experiments. Data for *lptB*$^+$, *lptB(L241Δ)* and *lptB-EHis$_8$* strains are also shown in Fig. S1C.

Genetic interactions between changes in R144, R198, and alterations to the CTD of LptB.

		Complements Δ*lptB*?[a]		Zone of inhibition[b]			
**Domains affected**	***lptB* allele**	**LB**	**Min**	**Bacitracin**	**Novobiocin**	**Erythromycin**	**Rifampin**
**None**	***lptB*⁺**	YES	YES	(8)	(8)	8 (10)	9 (10)
**Sig helix**	***lptB(R144H)***	NO	YES	N/A	N/A	N/A	N/A
**CT loop**	***lptB-EHis$_8$***	YES	YES	13	14 (22)	16 (20)	13
**Sig helix & CT loop**	***lptB(R144H)-EHis$_8$***	YES	YES	11	(9)	(16)	13
**CT loop**	***lptB(F239A)***	NO	NO	N/A	N/A	N/A	N/A
**Sig helix & CT loop**	***lptB(R144H/F239A)***	YES	YES	(8)	(8)	8 (10)	11
**CT loop**	***lptB(G236A)***	NO	NO	N/A	N/A	N/A	N/A
**Sig helix & CT loop**	***lptB(R144H/G236A)***	YES	YES	16	(9)	(20)	21
**CT helix**	***lptB(L235A)***	NO	NO	N/A	N/A	N/A	N/A
**Sig helix & CT helix**	***lptB(R144H/L235A)***	NO	NO	N/A	N/A	N/A	N/A
**CT helix**	***lptB(Y234A)***	NO	NO	N/A	N/A	N/A	N/A
**Sig helix & CT helix**	***lptB(R144H/Y234A)***	NO	NO	N/A	N/A	N/A	N/A
**Switch helix**	***lptB(R198E)***	NO	NO	N/A	N/A	N/A	N/A
**Sig helix & Switch helix**	***lptB(R144H/R198E)***	YES	YES	12	(9)	9 (16)	14

[a]Ability to complement a chromosomal Δ*lptB* allele on rich (LB) and minimal (Min) media.

[b]Antibiotic sensitivity was assessed by disc-diffusion assay on LB plates. Numbers represent the diameter (in mm) of partial (in parenthesis) or total zone of inhibition around the disc. Data is representative of at least three independent assays. N/A, not applicable because strain does not grow in LB.

Genetic interactions between changes in Walker A residue T45 and alterations to the CTD of LptB.

	Complements Δ*lptB*?[a]		Zone of inhibition (mm)[b]			
***lptB* allele**	**LB**	**Min**	**Bacitracin**	**Novobiocin**	**Erythromycin**	**Rifampin**
lptB^{+}	YES	YES	(8)	(8)	8 (12)	8
lptB(T45A)	YES	YES	(8)	(8)	8 (12)	8
lptB(F239A)	NO	NO	N/A	N/A	N/A	N/A
lptB(T45A/F239A)	YES	YES	14	13 (22)	14 (19)	12 (17)
lptB(Y234A)	NO	NO	N/A	N/A	N/A	N/A
lptB(T45A/Y234A)	NO	NO	N/A	N/A	N/A	N/A

[a]Ability to complement a chromosomal Δ*lptB* allele on rich (LB) and minimal (Min) media.

[b]Antibiotic sensitivity of viable haploid strains grown in LB using a disc diffusion assay. Numbers represent the diameter (in mm) of the zone of inhibition or that of partial growth (parenthesis). N/A, not applicable because strain does not grow in LB. Data are representative of at least three independent experiments.

Strains used in this study.

Strain	Genotype	Source
**DH5α**	F$^-$ *φ80lacZΔM15 Δ(lacZYA-argF)U169 recA1 endA1 hsdR17 (r_K^-, m_K^+) phoA supE44 λ$^-$ thi-1*	Life Technologies
**MC4100**	F$^-$ *araD139 Δ(argF-lac)U169 rpsL150 relA1 flbB5301 deoC1 ptsF25 rbsR*	(3)
**NR754**	MC4100 *ara$^+$*	(4)
**NR760**	NR754 *lptD4213*	(4)
**NR1768**	NR754 *lptB1-kan*	(5)
**NR1962**	NR754 *lptB1(G33C)-kan*	(5)
**NR1963**	NR754 *lptB1(R144H)-kan*	(5)
**NR2974**	NR754 *lptB1(T43S)-kan*	This study
**NR2975**	NR754 *lptB1(G33A)-kan*	This study
**NR2976**	NR754 *lptB1(I148T)-kan*	This study
**NR3025**	NR754 *lptB1(T45A)-kan*	This study
**NR3026**	NR754 *lptB1(L35Q)-kan*	This study
**NR3733**	NR754 *lptB1(S243Stop)-kan*	This study
**NR1872**	NR754 (pET23/42LptB-EHis8)	(6)
**NR2583**	NR754 (pET23/42LptB)	(6)
**NR2918**	NR754 (pET23/42LptBR198A-EHis8)	This study
**NR3085**	NR754 (pET23/42LptBR144A)	This study
**NR3086**	NR754 (pET23/42LptBR144A-EHis8)	This study
**NR3087**	NR754 (pET23/42LptBR144F)	This study
**NR3088**	NR754 (pET23/42LptBR144F-EHis8)	This study
**NR3314**	NR754 (pET23/42LptBR145A-EHis8)	This study
**NR3486**	NR754 (pET23/42LptBY234A)	This study
**NR3487**	NR754 (pET23/42LptBT45A/Y234A)	This study
**NR3488**	NR754 (pET23/42LptBR144H/Y234A)	This study
**NR4328**	NR754 (pET23/42LptBL235A)	This study
**NR4329**	NR754 (pET23/42LptBL235S)	This study
**NR4330**	NR754 (pET23/42LptBG236A)	This study
**NR4331**	NR754 (pET23/42LptBG236S)	This study
**NR4846**	NR754 (pET23/42LptBK177E)	This study
**NR4861**	NR754 (pET23/42LptBR198E)	This study
**NR4862**	NR754 (pET23/42LptBK177E-EHis8)	This study

**NR4863**	NR754 (pET23/42LptBR198E-EHis8)	This study
**NR4864**	NR754 (pET23/42LptB-RHis8)	This study
**NR5070**	NR754 (pET23/42LptBE199R)	This study
**NR5100**	NR754 (pET23/42LptBE199R-EHis8)	This study
**NR5153**	NR754 (pET23/42LptBF239A)	This study
**NR5235**	NR754 (pET23/42LptBD175A-EHis8)	This study
**NR2050**	NR754 Δ*lptB*::*frt tet2* (pRC7KanLptB)	(6)
**NR2093**	NR754 Δ*lptB*::*frt tet2* (pET23/42LptB-EHis8)	(6)
**NR2101**	NR754 Δ*lptB*::*frt tet2* (pET23/42LptB)	(6)
**NR2952**	NR754 Δ*lptB::frt tet2* (pET23/42LptBR198A)	This study
**NR3003**	NR754 Δ*lptB::frt tet2* (pET23/42LptBG33C)	This study
**NR3005**	NR754 Δ*lptB::frt tet2* (pET23/42LptBG33C-EHis8	This study
**NR3290**	NR754 Δ*lptB::frt tet2* (pET23/42LptBL35Q-EHis8	This study
**NR3291**	NR754 Δ*lptB::frt tet2* (pET23/42LptBT43A-EHis8	This study
**NR3292**	NR754 Δ*lptB::frt tet2* (pET23/42LptBT45A-EHis8	This study
**NR3032**	NR754 Δ*lptB::frt tet2* (pET23/42LptBR144H-EHis8)	This study
**NR4511**	NR754 Δ*lptB::frt tet2* (pET23/42LptBI148T-EHis8)	This study
**NR3234**	NR754 Δ*lptB::frt tet2* (pET23/42LptBR198A-EHis8	This study
**NR3294**	NR754 Δ*lptB::frt tet2* (pET23/42LptBLptBΔL241)	This study
**NR3302**	NR754 Δ*lptB::frt tet2* (pET23/42LptBI148S-EHis8)	This study
**NR3303**	NR754 Δ*lptB::frt tet2* (pET23/42LptBR144Q)	This study
**NR3304**	NR754 Δ*lptB::frt tet2* (pET23/42LptBR144Q-EHis8)	This study
**NR5243**	NR754 ΔlptB::frt tet2 (pET23/42LptBG33A)	This study
**NR3315**	NR754 Δ*lptB::frt tet2* (pET23/42LptBL35Q)	This study
**NR3316**	NR754 Δ*lptB::frt tet2* (pET23/42LptBT43S)	This study
**NR3317**	NR754 Δ*lptB::frt tet2* (pET23/42LptBT45A)	This study
**NR3324**	NR754 Δ*lptB::frt tet2* (pET23/42LptBR145A)	This study
**NR3325**	NR754 Δ*lptB::frt tet2* (pET23/42LptBI148S)	This study
**NR3533**	NR754 Δ*lptB::frt tet2* (pET23/42LptBT45A/F239A)	This study
**NR3534**	NR754 Δ*lptB::frt tet2* (pET23/42LptBR144H/F239A)	This study
**NR3657**	NR754 Δ*lptB::frt tet2* (pET23/42LptBS172A)	This study
**NR3658**	NR754 Δ*lptB::frt tet2* (pET23/42LptBS172A-EHis8)	This study
**NR4226**	NR754 Δ*lptB::frt tet2* (pET23/42LptBY234F)	This study
**NR4227**	NR754 Δ*lptB::frt tet2* (pET23/42LptBF239Y)	This study

**NR4345**	NR754 Δ*lptB::frt tet2* (pRC7KanLptB, pET23/42LptBR144H/L235A)	This study
**NR4388**	NR754 Δ*lptB::frt tet2* (pET23/42LptBR144H/G236A)	This study
**NR3174**	NR754 Δ*lptB::frt tet2* (pET23/42LptBR144H)	This study
**NR4512**	NR754 Δ*lptB::frt tet2* (pET23/42LptBI148V-EHis8)	This study
**NR4532**	NR754 Δ*lptB::frt tet2* (pET23/42LptBI148T)	This study
**NR4533**	NR754 Δ*lptB::frt tet2* (pET23/42LptBV230S)	This study
**NR4534**	NR754 Δ*lptB::frt tet2* (pET23/42LptBK231A)	This study
**NR4535**	NR754 Δ*lptB::frt tet2* (pET23/42LptBR232A)	This study
**NR4537**	NR754 Δ*lptB::frt tet2* (pET23/42LptBV233T)	This study
**NR4844**	NR754 Δ*lptB::frt tet2* (pET23/42LptBI148V)	This study
**NR4847**	NR754 Δ*lptB::frt tet2* (pET23/42LptBR240E)	This study
**NR4848**	NR754 Δ*lptB*::*frt tet2* (pET23/42LptB-R)	This study
**NR4849**	NR754 Δ*lptB*::*frt tet2* (pET23/42LptBI176T-EHis8)	This study
**NR4908**	NR754 Δ*lptB*::*frt tet2* (pET23/42LptBK177A)	This study
**NR4912**	NR754 Δ*lptB*::*frt tet2* (pET23/42LptBK177A-EHis8)	This study
**NR4913**	NR754 Δ*lptB*::*frt tet2* (pET23/42LptBE199A)	This study
**NR4914**	NR754 Δ*lptB*::*frt tet2* (pET23/42LptBR144H/R198)	This study
**NR4915**	NR754 Δ*lptB*::*frt tet2* (pET23/42LptBE199A-EHis8)	This study
**NR4916**	NR754 Δ*lptB*::*frt tet2* (pET23/42LptBE237A)	This study
**NR4918**	NR754 Δ*lptB*::*frt tet2* (pET23/42LptBD238A)	This study
**NR5059**	NR754 Δ*lptB*::*frt tet2* (pET23/42LptBI176T)	This study
**NR5233**	NR754 Δ*lptB*::*frt tet2* (pET23/42LptBD175A)	This study
**NR5110**	NR754 Δ*lptB::frt tet2* (pRC7KanLptB, pET23/42LptBR144H/E163Q)	This study
**NR5969**	NR754 Δ*lptB::frt tet2* (pRC7KanLptB, pET23/42LptBR144H/E163Q/F239A)	This study
**NR5112**	NR754 ΔlptB::frt tet2 (pRC7KanLptB, pET23/42LptBE163Q/F239A)	This study
**NR2723**	NR754 (pET23/42LptBE163Q)	(6)

Primers used in this study.

Primer name	Change	Primer sequence 5' to 3'
**T7 seq**	N/A	TAA TAC GAC TCA CTA TAG
**5LptB77up**	N/A	GTT CTG GTG CCG TCG CAG
**3LptB50down**	N/A	CAT GTT CAG AAT CGT ACT CTC CTG C
**LptBG33Asense**	G33A	GGG GAA ATT GTC GCT CTG CTG GGG C
**LptBG33Aanti**	G33A	GCC CCA GCA GAG CGA CAA TTT CCC C
**5LptBG33C**	G33C	CGG GGA AAT TGT CTG TCT GCT GGG G
**3LptBG33C**	G33C	CCC CAG CAG ACA GAC AAT TTC CCC G
**LptB_L35Q**	L35Q	CAC CGT TTG GCC CCT GCA GAC CGA C
**LptB_L35Q_antisense**	L35Q	GTC GGT CTG CAG GGG CCA AAC GGT G
**LptB_T43S**	T43S	CAT GTA GAA AGT GGT GCT CTT ACC GGC ACC G
**LptB_T43S_antisense**	T43S	CGG TGC CGG TAA GAG CAC CAC TTT CTA CAT G
**LptB_T45A**	T45A	GTG CCG GTA AGA CCA CCG CTT TCT ACA TGG TTG
**LptB_T45A_antisense**	T45A	CAA CCA TGT AGA AAG CGG TGG TCT TAC CGG CAC
**lptB R144A sense**	R144A	GGG GTG AAC GTG CCC GTG TAG AAA T
**lptB R144A anti**	R144A	ATT TCT ACA CGG GCA CGT TCA CCC C
**lptB R144F sense**	R144F	CTC CGG GGG TGA ACG TTT CCG TGT AGA A
**lptB R144F anti**	R144F	TTC TAC ACG GAA ACG TTC ACC CCC GGA G
**5LptBR144H**	R144H	GGG TGA ACG TCA CCG TGT AGA AAT TG
**3LptBR144H**	R144H	CAA TTT CTA CAC GGT GAC GTT CAC CC
**lptBR144Q sense**	R144Q	GGG GGT GAA CGT CAA CGT GTA GAA ATT GCC
**lptBR144Q anti**	R144Q	GGC AAT TTC TAC ACG TTG ACG TTC ACC CCC
**LptB_R145A**	R145A	GGT GAA CGT CGC GCT GTA GAA ATT GCC CG
**LptB_R145A_antisense**	R145A	CGG GCA ATT TCT ACA GCG CGA CGT TCA CC
**LptB_I148S**	I148S	GTC GCC GTG TAG AAA GTG CCC GCG C
**LptB_I148S_antisense**	I148S	GCG CGG GCA CTT TCT ACA CGG CGA C
**LptBI148Tsense**	I148T	GTC GCC GTG TAG AAA CTG CCC GCG CAC TGG

**LptBI148Tanti**	I148T	CCA GTG CGC GGG CAG TTT CTA CAC GGC GAC
**LptBI148Vsense**	I148V	GTC GCC GTG TAG AAG TTG CCC GCG CAC TGG
**LptBI148Vanti**	I148V	CCA GTG CGC GGG CAA CTT CTA CAC GGC GAC
**LptBS172Asense**	S172A	GGG TTG ACC CGA TCG CAG TTA TCG ACA TTA AAC
**LptBS172Aanti**	S172A	GTT TAA TGT CGA TAA CTG CGA TCG GGT CAA CCC
**LptBD175Asense**	D175A	CCG ATC TCG GTT ATC GCC ATT AAA CGC ATC
**LptBD175Aanti**	D175A	GAT GCG TTT AAT GGC GAT AAC CGA GAT CGG
**LptB I176T Sense**	I176T	CTC GGT TAT CGA CAC TAA ACG CAT C
**LptB I176T Anti**	I176T	GAT GCG TTT AGT GTC GAT AAC CGA G
**LptBK177Asense**	K177A	CGG TTA TCG ACA TTG CAC GCA TCA TTG AGC
**LptBK177Aanti**	K177A	GCT CAA TGA TGC GTG CAA TGT CGA TAA CCG
**LptBK177Esense**	K177E	CGG TTA TCG ACA TTG AAC GCA TCA TTG AGC
**LptBK177Eanti**	K177E	GCT CAA TGA TGC GTT CAA TGT CGA TAA CCG
**5LptBR198A**	R198A	CTG ACC ACA ACG TGG CGG AAA CAC TGG CG
**3LptBR198A**	R198A	CGC CAG TGT TTC CGC CAC GTT GTG GTC AG
**LptBR198Esense**	R198E	CTG ACC ACA ACG TGG AGG AAA CAC TGG CGG
**LptBR198Eanti**	R198E	CCG CCA GTG TTT CCT CCA CGT TGT GGT CAG
**LptBE199Asense**	E199A	CCA CAA CGT GCG TGC TAC ACT GGC GG
**LptBE199Aanti**	E199A	CCG CCA GTG TAG CAC GCA CGT TGT GG
**LptBE199Rsense**	E199R	CCA CAA CGT GCG TCG TAC ACT GGC GG
**LptBE199Ranti**	E199R	CCG CCA GTG TAC GAC GCA CGT TGT GG
**LptBV230Ssense**	V230S	CAA GAC GAA CAC AGC AAG CGT GTA TAC CTT GGG G
**LptBV230Santi**	V230S	CCC CAA GGT ATA CAC GCT TGC TGT GTT CGT CTT G
**LptBK231Asense**	K231A	CAA GAC GAA CAC GTT GCC CGT GTA TAC CTT GGG
**LptBK231Aanti**	K231A	CCC AAG GTA TAC ACG GGC AAC GTG TTC GTC TTG

**LptBR232Asense**	R232A	GAC GAA CAC GTT AAG GCT GTA TAC CTT GGG G
**LptBR232Aanti**	R232A	CCC CAA GGT ATA CAG CCT TAA CGT GTT CGT C
**LptBV233T**	V233T	CAA GAC GAA CAC GTT AAG CGT ACC TAC CTT GGG GAA GAC TTC AGA
**LptBV233T_antisense**	V233T	TCT GAA GTC TTC CCC AAG GTA GGT ACG CTT AAC GTG TTC GTC TTG
**5LptB77up**	N/A	GTT CTG GTG CCG TCG CAG
**3LptB50down**	N/A	CAT GTT CAG AAT CGT ACT CTC CTG C
**LptBG33Asense**	G33A	GGG GAA ATT GTC GCT CTG CTG GGG C
**LptBG33Aanti**	G33A	GCC CCA GCA GAG CGA CAA TTT CCC C
**5LptBG33C**	G33C	CGG GGA AAT TGT CTG TCT GCT GGG G
**3LptBG33C**	G33C	CCC CAG CAG ACA GAC AAT TTC CCC G
**LptB_L35Q**	L35Q	CAC CGT TTG GCC CCT GCA GAC CGA C
**LptB_L35Q_antisense**	L35Q	GTC GGT CTG CAG GGG CCA AAC GGT G
**LptB_T43S**	T43S	CAT GTA GAA AGT GGT GCT CTT ACC GGC ACC G
**LptB_T43S_antisense**	T43S	CGG TGC CGG TAA GAG CAC CAC TTT CTA CAT G
**LptB_T45A**	T45A	GTG CCG GTA AGA CCA CCG CTT TCT ACA TGG TTG
**LptB_T45A_antisense**	T45A	CAA CCA TGT AGA AAG CGG TGG TCT TAC CGG CAC
**lptB R144A sense**	R144A	GGG GTG AAC GTG CCC GTG TAG AAA T
**lptB R144A anti**	R144A	ATT TCT ACA CGG GCA CGT TCA CCC C
**lptB R144F sense**	R144F	CTC CGG GGG TGA ACG TTT CCG TGT AGA A
**lptB R144F anti**	R144F	TTC TAC ACG GAA ACG TTC ACC CCC GGA G
**5LptBR144H**	R144H	GGG TGA ACG TCA CCG TGT AGA AAT TG
**3LptBR144H**	R144H	CAA TTT CTA CAC GGT GAC GTT CAC CC
**lptBR144Q sense**	R144Q	GGG GGT GAA CGT CAA CGT GTA GAA ATT GCC
**lptBR144Q anti**	R144Q	GGC AAT TTC TAC ACG TTG ACG TTC ACC CCC
**LptB_R145A**	R145A	GGT GAA CGT CGC GCT GTA GAA ATT GCC CG
**LptB_R145A_antisense**	R145A	CGG GCA ATT TCT ACA GCG CGA CGT TCA CC
**LptB_I148S**	I148S	GTC GCC GTG TAG AAA GTG CCC GCG C

**LptB_I148S_antisense**	I148S	GCG CGG GCA CTT TCT ACA CGG CGA C
**LptBI148Tsense**	I148T	GTC GCC GTG TAG AAA CTG CCC GCG CAC TGG
**LptBI148Tanti**	I148T	CCA GTG CGC GGG CAG TTT CTA CAC GGC GAC
**LptBI148Vsense**	I148V	GTC GCC GTG TAG AAG TTG CCC GCG CAC TGG
**LptBI148Vanti**	I148V	CCA GTG CGC GGG CAA CTT CTA CAC GGC GAC
**LptBS172Asense**	S172A	GGG TTG ACC CGA TCG CAG TTA TCG ACA TTA AAC
**LptBS172Aanti**	S172A	GTT TAA TGT CGA TAA CTG CGA TCG GGT CAA CCC
**LptBD175Asense**	D175A	CCG ATC TCG GTT ATC GCC ATT AAA CGC ATC
**LptBD175Aanti**	D175A	GAT GCG TTT AAT GGC GAT AAC CGA GAT CGG
**LptB I176T Sense**	I176T	CTC GGT TAT CGA CAC TAA ACG CAT C
**LptB I176T Anti**	I176T	GAT GCG TTT AGT GTC GAT AAC CGA G
**LptBK177Asense**	K177A	CGG TTA TCG ACA TTG CAC GCA TCA TTG AGC
**LptBK177Aanti**	K177A	GCT CAA TGA TGC GTG CAA TGT CGA TAA CCG
**LptBK177Esense**	K177E	CGG TTA TCG ACA TTG AAC GCA TCA TTG AGC
**LptBK177Eanti**	K177E	GCT CAA TGA TGC GTT CAA TGT CGA TAA CCG
**5LptBR198A**	R198A	CTG ACC ACA ACG TGG CGG AAA CAC TGG CG
**3LptBR198A**	R198A	CGC CAG TGT TTC CGC CAC GTT GTG GTC AG
**LptBR198Esense**	R198E	CTG ACC ACA ACG TGG AGG AAA CAC TGG CGG
**LptBR198Eanti**	R198E	CCG CCA GTG TTT CCT CCA CGT TGT GGT CAG
**LptBE199Asense**	E199A	CCA CAA CGT GCG TGC TAC ACT GGC GG
**LptBE199Aanti**	E199A	CCG CCA GTG TAG CAC GCA CGT TGT GG
**LptBE199Rsense**	E199R	CCA CAA CGT GCG TCG TAC ACT GGC GG
**LptBE199Ranti**	E199R	CCG CCA GTG TAC GAC GCA CGT TGT GG
**LptBV230Ssense**	V230S	CAA GAC GAA CAC AGC AAG CGT GTA TAC CTT GGG G
**LptBV230Santi**	V230S	CCC CAA GGT ATA CAC GCT TGC TGT GTT CGT CTT G

**LptBK231Asense**	K231A	CAA GAC GAA CAC GTT GCC CGT GTA TAC CTT GGG
**LptBK231Aanti**	K231A	CCC AAG GTA TAC ACG GGC AAC GTG TTC GTC TTG
**LptBR232Asense**	R232A	GAC GAA CAC GTT AAG GCT GTA TAC CTT GGG G
**LptBR232Aanti**	R232A	CCC CAA GGT ATA CAG CCT TAA CGT GTT CGT C
**LptBV233T**	V233T	CAA GAC GAA CAC GTT AAG CGT ACC TAC CTT GGG GAA GAC TTC AGA
**LptBV233T_antisense**	V233T	TCT GAA GTC TTC CCC AAG GTA GGT ACG CTT AAC GTG TTC GTC TTG
**LptBY234A_antisense**	Y234A	TGA AGT CTT CCC CAA GGG CTA CAC GCT TAA CGT GTT CGT C
**LptBY234Fsense**	Y234F	CACGTTAAGCGTGTATTCCTTGGGGAAGA CTTC
**LptBY234Fanti**	Y234F	GAA GTC TTC CCC AAG GAA TAC ACG CTT AAC GTG
**LptBL235Asense**	L235A	GTT AAG CGT GTA TAC GCA GGG GAA GAC TTC AG
**LptBL235Aanti**	L235A	CTG AAG TCT TCC CCT GCG TAT ACA CGC TTA AC
**LptBL235Ssense**	L235S	GTT AAG CGT GTA TAC AGT GGG GAA GAC TTC AG
**LptBL235Santi**	L235S	CTG AAG TCT TCC CCA CTG TAT ACA CGC TTA AC
**LptBG236Asense**	G236A	GCG TGT ATA CCT TGC AGA AGA CTT CAG ACT C
**LptBG236Aanti**	G236A	GAG TCT GAA GTC TTC TGC AAG GTA TAC ACG C
**LptBG236Ssense**	G236S	GCG TGT ATA CCT TAG CGA AGA CTT CAG ACT C
**LptBG236Santi**	G236S	GAG TCT GAA GTC TTC GCT AAG GTA TAC ACG C
**LptBE237A**	E237A	GCG TGT ATA CCT TGG GGC GGA CTT CAG ACT CTG ACC
**LptBE237A_antisense**	E237A	GGT CAG AGT CTG AAG TCC GCC CCA AGG TAT ACA CGC
**5LptBD238A**	D238A	GTG TAT ACC TTG GGG AAG CGT TCA GAC TCT GAC CTA GG
**3LptBD238A**	D238A	CCT AGG TCA GAG TCT GAA CGC TTC CCC AAG GTA TAC AC
**LptBF239A**	F239A	CTT GGG GAA GAC GCC AGA CTC TGA GAG CAC
**LptBF239A_antisense**	F239A	GTG CTC TCA GAG TCT GGC GTC TTC CCC AAG

**LptBF239Ysense**	F239Y	CCT TGG GGA AGA CTA CAG ACT CTG AGA GCA C
**LptBF239Yanti**	F239Y	GTG CTC TCA GAG TCT GTA GTC TTC CCC AAG G
**LptBR240E**	R240E	ACC TTG GGG AAG ACT TCG AAC TCT GAC CTA GGC TGC
**LptBR240E_antisense**	R240E	GCA GCC TAG GTC AGA GTT CGA AGT CTT CCC CAA GGT
**LptB_241Amber**	ΔL241	GGG AAG ACT TCA GAT AGT GAG AGC ACC AC
**LptB_241Amber_antisense**	ΔL241	GTG GTG CTC TCA CTA TCT GAA GTC TTC CC
**LptB-R242sense**	lptB-R	GGA AGA CTT CAG ACT CCG TTA GCA CCA CCA CCA CCA C
**LptB-R242anti**	lptB-R	GTG GTG GTG GTG GTG CTA ACG GAG TCT GAA GTC TTC C
**LptBR-His8sense**	lptB-RHis8	GAA GAC TTC AGA CTC CGT CAC CAC CAC CAC CAC
**LptBR-His8anti**	lptB-RHis8	GTG GTG GTG GTG GTG ACG GAG TCT GAA GTC TTC
**His8TEV-LptB-fwd**	His8-LptB	CAT CAC CAT CAC GAA AAC CTG TAC TTC CAG GGC GCA AGC GCA ACA TTA ACT G
**His8TEV-LptB-rev**	His8-LptB	GCT TGG CCC TGG AAG TAC AGG TTT TCG TGA TGG TGA TGA TGA TGA TGA TGA CCC ATG G
