# Supplementary material for: Combining Mutations That Inhibit Two Distinct Steps of the ATP Hydrolysis Cycle Restores Wild-Type Function in the Lipopolysaccharide Transporter and Shows that ATP Binding Triggers Transport
Source: mBio. 2019 Aug 20;10(4):e01931-19. doi: 10.1128/mBio.01931-19 (PMC6703430; doi:10.1128/mBio.01931-19)
Supplement: FIG S1 [file mBio.01931-19-sf001.pdf]

A

Allele	Allele location	Protein	CT extension after residue L241	Haploid phenotype
lptB⁺	chromosome	N-LptB-C	None	Wild-type
lptB-His	chromosome	N-LptB-C	-RGSHHHHHHTD	Unable to generate strain
lptB1	chromosome	N-LptB-C	-ISPSPSPYGSGPEAKCRLELLRS… …SYSLESIGTSE	Increased OM permeability
lptB⁺	plasmid	N-LptB-C	None	Wild-type
lptB-EHis$_8$	plasmid	N-LptB-C	-EHHHHHHHH	Increased OM permeability
lptB-R	plasmid	N-LptB-C	-R	Increased OM permeability
lptB-RHis$_8$	plasmid	N-LptB-C	-RHHHHHHHH	Lethal

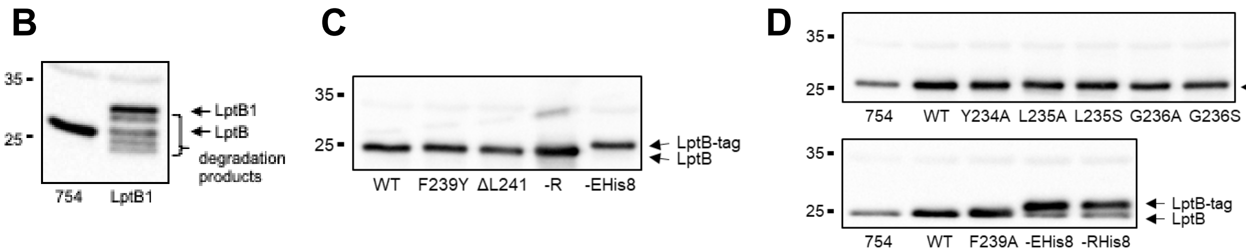

E

lptB allele	Complements Δ*lptB*?
lptB(Y234A)	NO
lptB(Y234F)	YES
lptB(L235A)	NO
lptB(L235S)	NO
lptB(G236A)	NO
lptB(G236S)	NO
lptB(F239A)	NO
lptB(F239Y)	YES
lptB(ΔL241)	YES

F

	Zone of inhibition			
***lptB* allele**	**Bacitracin**	**Novobiocin**	**Erythromycin**	**Rifampin**
754, *lptB*⁺	<6	<6	(10)	9
lptB1	15	19 (24)	16 (21)	16
lptB⁺	(8)	(8)	8 (10)	9 (10)
lptB(F239Y)	8	10 (12)	10 (13)	11
lptB(ΔL241)	11	14 (21)	13 (16)	12
lptB-R	8	14 (19)	13 (16)	13
lptB-EHis$_8$	13	14 (22)	16 (20)	13

Figure S1: Altering the CTD of LptB results in functional defects. (A) The addition of CT extensions to LptB disrupts activity to varying degree. Summary of functional analyses for various *lptB* haploid strains: When investigating why some CT-tagged LptB variants could support growth while others could not, we noticed the two types significantly differed at residue 242, which is located in the linker between the last residue (L241) of wild-type LptB and the tag. The lethal chromosomal *lptB-His* allele encodes a positively charged arginine, while the complementing plasmid-encoded *lptB-EHis$_8$* and chromosomal *lptB1* alleles encode a negatively charged glutamate and a neutral isoleucine, respectively. To examine if the addition of an arginine after the native L241 residue affected LptB function on its own or in the context of the His-tag, we constructed plasmids bearing *lptB* alleles encoding LptB followed by only an arginine (*lptB-R*) or an arginine followed by a poly-histidine tag (*lptB-RHis$_8$*) and compared their function to that of wild-type *lptB* (*lptB*⁺) and *lptB-EHis$_8$*. The *lptB-R* and *lptB-EHis$_8$* alleles complemented chromosomal Δ*lptB*, but the resulting haploid strains are sensitive to hydrophobic antibiotics, indicating these strains, like an *lptB1* strain, are defective in LPS transport. In contrast, the *lptB-RHis$_8$* allele could not complement chromosomal Δ*lptB* despite producing as much protein as an *lptB-EHis$_8$* strain (see below). Thus, adding an arginine or oligopeptide to the CTD of LptB causes partial loss-of-function defects, while the combination of an arginine and oligopeptide is lethal. **(B)** LptB immunoblot comparing strains that chromosomally encode LptB^{WT}, labeled 754, or LptB1. **(C)** LptB immunoblot of samples from haploid strains encoding LptB CTD variants on the pET23/42-LptB plasmid. WT refers to haploid strain NR2101, which produces LptB^{WT} from pET23/42-LptB. **(D)** LptB immunoblot of samples from merodiploid strains producing LptB CTD variants from the pET23/42-LptB plasmid and LptB^{WT} from the native *lptB* locus. 754 refers to NR754, the wild-type strain with *lptB*⁺ at the native locus. WT refers to strain NR2583, which produces LptB^{WT} from both the chromosome and pET23/42-LptB. Data are representative of at least three independent experiments. **(E)** Ability of CTD *lptB* alleles to complement a chromosomal Δ*lptB* allele in rich (LB) medium. **(F)** OM permeability of haploid strains producing LptB CTD variants was determined by disc diffusion assay. All alleles, except *lptB*⁺ in NR754 and *lptB1* which are located on the chromosome, are encoded on the pET23/42-LptB plasmid. Increased sensitivity was reproducibly observed for strains shown indicative of LPS transport defects compared to wild-type *lptB*⁺ strain NR2101, while those not shown (containing changes V230S, K231A, R232A, V233T, E237A, D238A, F239Y, and R240A) behaved like the wild-type strain NR2101. Numbers represent the diameter (in mm) of the zone of inhibition or that of partial growth (parenthesis) around a 6-mm disc containing an antibiotic.
