# Supplementary material for: Combining Mutations That Inhibit Two Distinct Steps of the ATP Hydrolysis Cycle Restores Wild-Type Function in the Lipopolysaccharide Transporter and Shows that ATP Binding Triggers Transport
Source: mBio. 2019 Aug 20;10(4):e01931-19. doi: 10.1128/mBio.01931-19 (PMC6703430; doi:10.1128/mBio.01931-19)
Supplement: TEXT S1 [file mBio.01931-19-s0001.pdf]

Combining mutations that inhibit two distinct steps of the ATP hydrolysis cycle restores wild-type function in the lipopolysaccharide transporter and shows that ATP binding triggers transport

Brent W. Simpson, Karanbir S. Pahil, Tristan W. Owens, Emily A. Lundstedt, Rebecca M. Davis, Daniel Kahne, and Natividad Ruiz

SUPPLEMENTAL REFERENCES FOR FIG. S2 AND DATASET 1.

1. Finn RD, Tate J, Mistry J, Coggill PC, Sammut SJ, Hotz HR, Ceric G, Forslund K, Eddy SR, Sonnhammer EL, Bateman A. 2008. The Pfam protein families database. Nucleic Acids Res 36:D281-8.
2. Waterhouse AM, Procter JB, Martin DM, Clamp M, Barton GJ. 2009. Jalview Version 2--a multiple sequence alignment editor and analysis workbench. Bioinformatics 25:1189-91.
3. Casadaban MJ. 1976. Transposition and fusion of the lac genes to selected promoters in *Escherichia coli* using bacteriophage lambda and Mu. J Mol Biol 104:541-55.
4. Ruiz N, Wu T, Kahne D, Silhavy TJ. 2006. Probing the barrier function of the outer membrane with chemical conditionality. ACS Chem Biol 1:385-95.
5. May JM, Owens TW, Mandler MD, Simpson BW, Lazarus MB, Sherman DJ, Davis RM, Okuda S, Massefski W, Ruiz N, Kahne D. 2017. The antibiotic novobiocin binds and activates the ATPase that powers lipopolysaccharide transport. J Am Chem Soc 139:17221-17224.
6. Sherman DJ, Lazarus MB, Murphy L, Liu C, Walker S, Ruiz N, Kahne D. 2014. Decoupling catalytic activity from biological function of the ATPase that powers lipopolysaccharide transport. Proc Natl Acad Sci U S A 111:4982-7.
