# Supplementary material for: Combining Mutations That Inhibit Two Distinct Steps of the ATP Hydrolysis Cycle Restores Wild-Type Function in the Lipopolysaccharide Transporter and Shows that ATP Binding Triggers Transport
Source: mBio. 2019 Aug 20;10(4):e01931-19. doi: 10.1128/mBio.01931-19 (PMC6703430; doi:10.1128/mBio.01931-19)
Supplement: FIG S3 [file mBio.01931-19-sf003.pdf]

A

	His_8-$LptB^{E163Q}$ - ATP
**Space Group**	$C222_1$
**Unit Cell**	
**Dimensions (a, b, c), Å**	65.36, 137.59, 100.97
**Angles (α, β, γ), °**	90, 90, 90
**Data Collection**	
**Resolution range, Å**	100.97 - 1.96 (2.01 - 196)
**Completeness, %**	99.2 (94.8)
**R_{merge}**	0.114 (1.476)
**Mean I/σ(I)**	9.4 (0.9)
**Multiplicity**	3.6 (3.3)
**$CC_{1/2}$**	0.997 (0.381)
**Refinement**	
**No. reflections**	32755
**Rwork, % / Rfree, %**	18.7, 0.219
**Average B ($Å^2$) protein**	32.7
**Average B ($Å^2$) ligands**	26.9
**Average B ($Å^2$) waters**	36.6
**Ramachandran plot**	
**Favored/disallowed (%)**	98.33 / 0.00
**rmsd from ideal geometry**	
**Bond lengths, Å**	0.0042
**Bond angles, °**	0.67
**PDB code**	6MBN

B

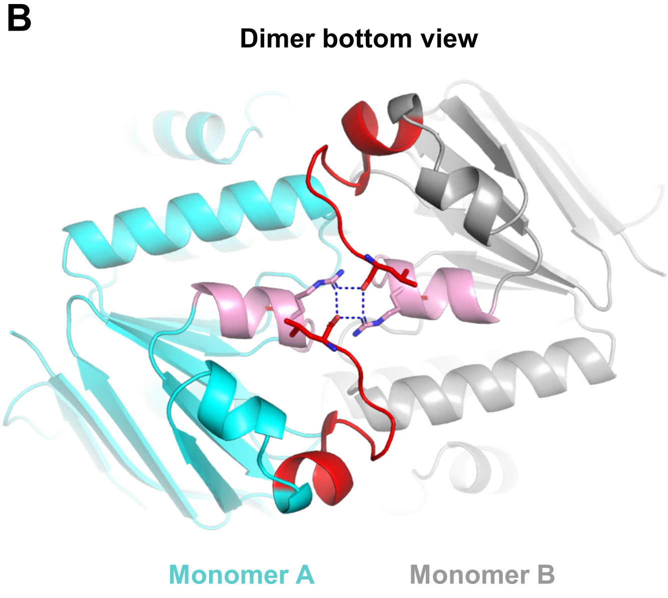

C

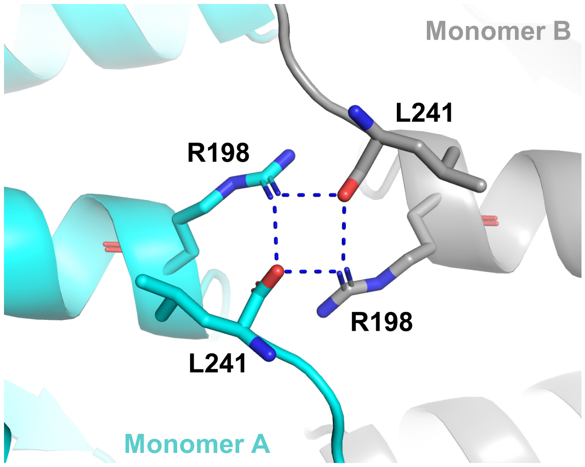

Figure S3: The CTD of LptB mediates intra- and inter-monomer contacts through interactions with the switch helix. (A) Data collection and refinement statistics of PBD 6MBN structure of the ATP-bound His_8-$LptB^{E163Q}$ dimer. **(B)** Cartoon representation of the ATP-bound His_8-$LptB^{E163Q}$ dimer (PBD 6MBN) showing that the carboxylic acid of the last residue in LptB, L241, forms ionic pairs (blue dotted lines) with residue R198 in the switch helix both within each monomer and across monomers. A bottom view of the LptB dimer in which LptB monomers are colored cyan (monomer A) and light grey (monomer B), the switch helix containing R198 is colored pink, and the CTD is colored red. R198 and L241 are shown as sticks. **(C)** Detailed view of the interactions between residues R198 and L241. LptB monomers are colored cyan (monomer A) and light grey (monomer B).
