# Supplementary material for: Combining Mutations That Inhibit Two Distinct Steps of the ATP Hydrolysis Cycle Restores Wild-Type Function in the Lipopolysaccharide Transporter and Shows that ATP Binding Triggers Transport
Source: mBio. 2019 Aug 20;10(4):e01931-19. doi: 10.1128/mBio.01931-19 (PMC6703430; doi:10.1128/mBio.01931-19)
Supplement: FIG S4 [file mBio.01931-19-sf004.pdf]

A

	Zone of inhibition			
***lptB* allele**	**Bacitracin**	**Novobiocin**	**Erythromycin**	**Rifampin**
***lptB*$^+$**	<6	<6	(10)	9
lptB1	15	19 (24)	16 (21)	16
lptB1(G33A)	<6	<6	(10)	9
lptB1(G33C)	<6	<6	(10)	9
lptB1(L35Q)	<6	<6	(11)	11
lptB1(T43S)	<6	<6	(10)	9
lptB1(T45A)	<6	<6	(11)	10
lptB1(R144H)	19	<6	(21)	24
lptB1(I148T)	<6	<6	(10)	9
lptB1(S243Stop)	<6	<6	(10)	9

B

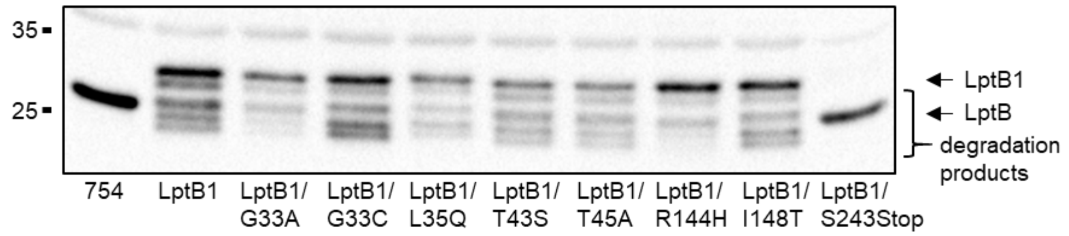

C

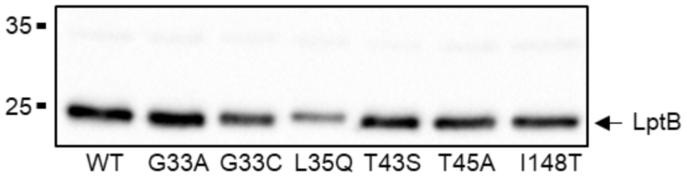

D

	Zone of inhibition			
***lptB* allele**	**Bacitracin**	**Novobiocin**	**Erythromycin**	**Rifampin**
***lptB*$^+$**	<6	<6	(12)	10
lptB-EHis$_8$	13	15 (23)	15 (19)	14
lptB(G33C)-EHis$_8$	<6	<6	(12)	10
lptB(L35Q)-EHis$_8$	<6	<6	(11)	11
lptB(T43S)-EHis$_8$	<6	<6	(10)	10
lptB(T45A)-EHis$_8$	<6	<6	(11)	10
lptB(R144H)-EHis$_8$	10	<6	(14)	13
lptB(I148T)-EHis$_8$	<6	<6	(11)	10

Figure S4: Structure-function analysis of *lptB1* suppressors. OM-permeability assay (**A**) and LptB immunoblots (**B**) of chromosomally-encoded *lptB1* and suppressors performed as described in Fig. S1 and methods. *lptB*$^+$ refers to strain NR754. (**C)** LptB immunoblot of samples from haploid strains producing LptB variants from pET23/42LptB derivatives, as described in Fig. S1. **(D)** OM-permeability assay of haploid cells carrying pET23/42LptB-EHis$_8$ derivatives with the substitutions we found as suppressors of *lptB1*. Assays were performed as described in Fig. S1. Data are representative of at least three independent experiments.
