# Supplementary material for: Combining Mutations That Inhibit Two Distinct Steps of the ATP Hydrolysis Cycle Restores Wild-Type Function in the Lipopolysaccharide Transporter and Shows that ATP Binding Triggers Transport
Source: mBio. 2019 Aug 20;10(4):e01931-19. doi: 10.1128/mBio.01931-19 (PMC6703430; doi:10.1128/mBio.01931-19)
Supplement: FIG S5 [file mBio.01931-19-sf005.pdf]

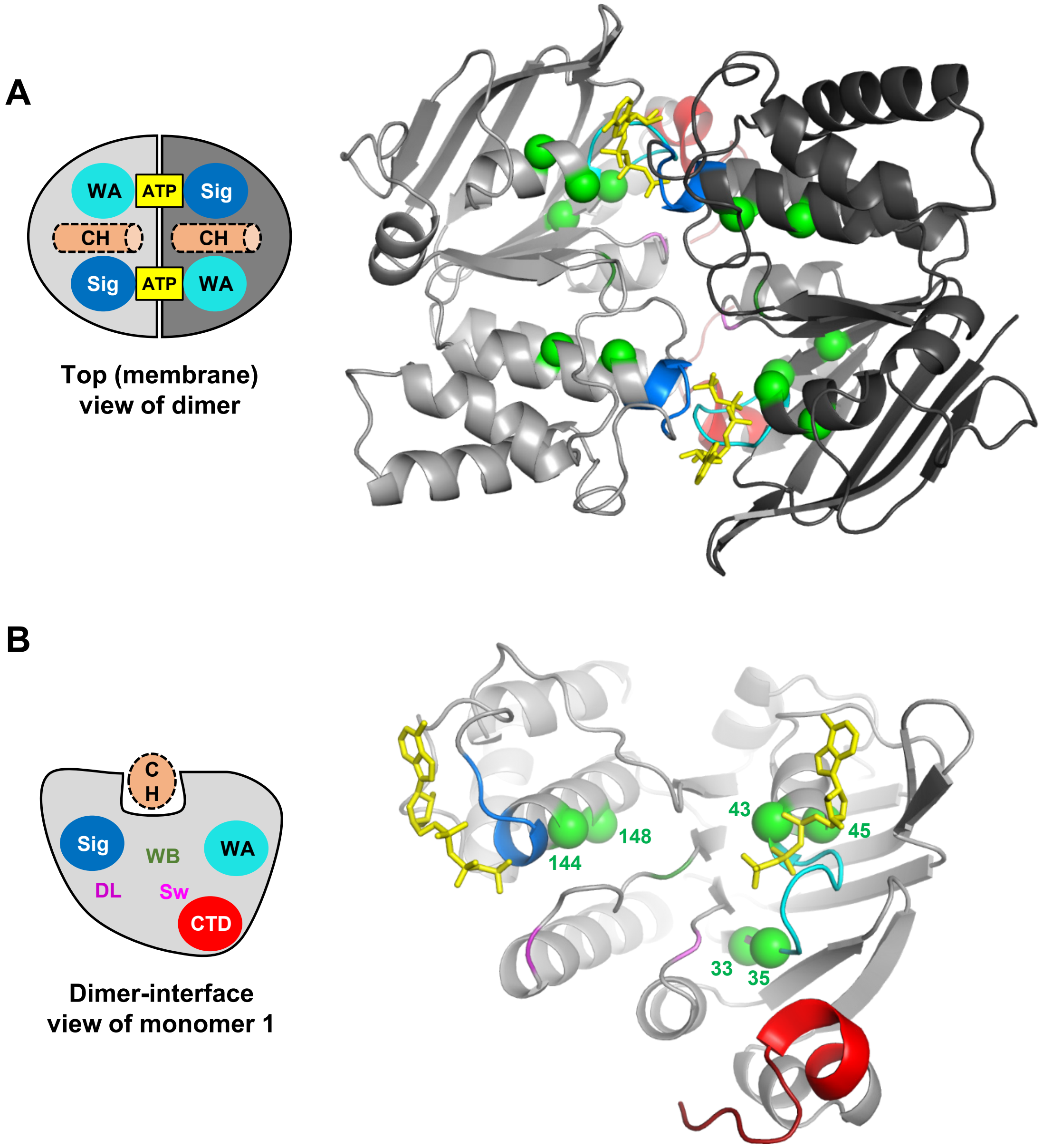

Figure S5: Location of *lptB1* suppressors. Cartoon representation of the structure an His$_8$-LptB^{E163Q} dimer (**A**) and monomer (**B**) showing the location of the suppressors of *lptB1* (represented as forest green spheres). The Walker A (cyan), signature motif (marine), and CTD (red) are colored in the X-ray structure. ATP is shown in yellow sticks. The location of additional motifs important for function of ABC transporters is also shown on the cartoons on the left: WB marks the Walker B motif, DL the D loop, Sw the switch loop, and CH a coupling helix of either LptF or LptG. Numbers in green shown in **(B)** represent the residue number altered in the suppressors.
