# Supplementary material for: Combining Mutations That Inhibit Two Distinct Steps of the ATP Hydrolysis Cycle Restores Wild-Type Function in the Lipopolysaccharide Transporter and Shows that ATP Binding Triggers Transport
Source: mBio. 2019 Aug 20;10(4):e01931-19. doi: 10.1128/mBio.01931-19 (PMC6703430; doi:10.1128/mBio.01931-19)
Supplement: FIG S2 [file mBio.01931-19-sf002.pdf]

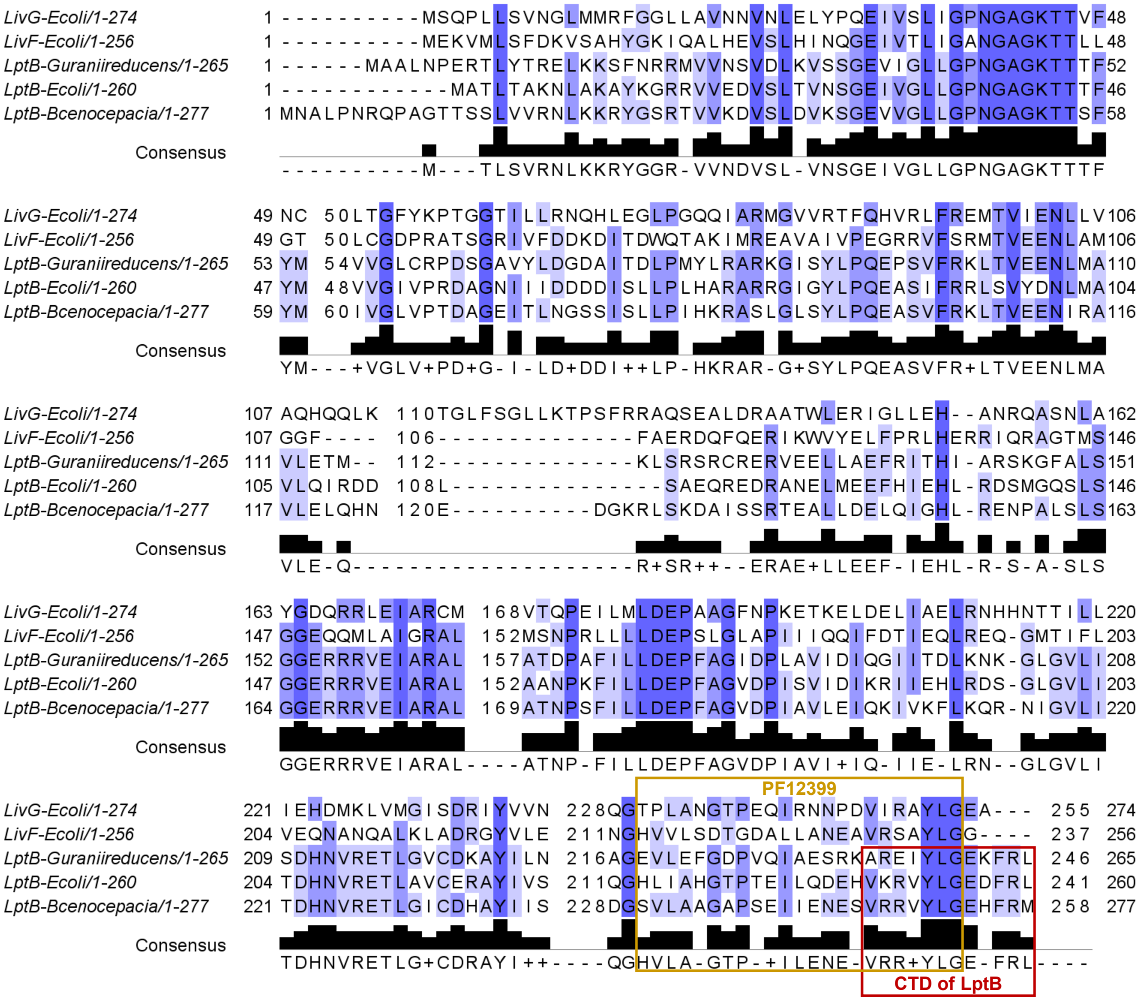

Figure S2: Sequence alignment of LptB and the ATPases of branched-chain amino acid transporters. Clustal W alignment of LptB homologs with closely related branched-chain amino acid transporter ATPases LivF/G shows that they share homology at their CTDs. The protein family PF12399 is a conserved sequence found in both protein families and ending in a characteristic YLG sequence (1). The CTD of LptB has additional conserved residues not found in PF12399. Colored using the percentage identity color scheme in Jalview (2). Light to dark purple indicates 50 to 100% identity respectively and white indicates <50% identities. Protein sequences aligned were LivF (WP_000416891.1), LivG (WP_136807488.1), and LptB (WP_000224099.1) from *E. coli* K12 substr. MG1655 and LptB homologs from *Geobacter uraniireducens* Rfr and *Burkholderia cenocepacia* (WP_011939814.1 and WP_124633598.1, respectively).
