# Supplementary material for: Combining Mutations That Inhibit Two Distinct Steps of the ATP Hydrolysis Cycle Restores Wild-Type Function in the Lipopolysaccharide Transporter and Shows that ATP Binding Triggers Transport
Source: mBio. 2019 Aug 20;10(4):e01931-19. doi: 10.1128/mBio.01931-19 (PMC6703430; doi:10.1128/mBio.01931-19)
Supplement: FIG S7 [file mBio.01931-19-sf007.pdf]

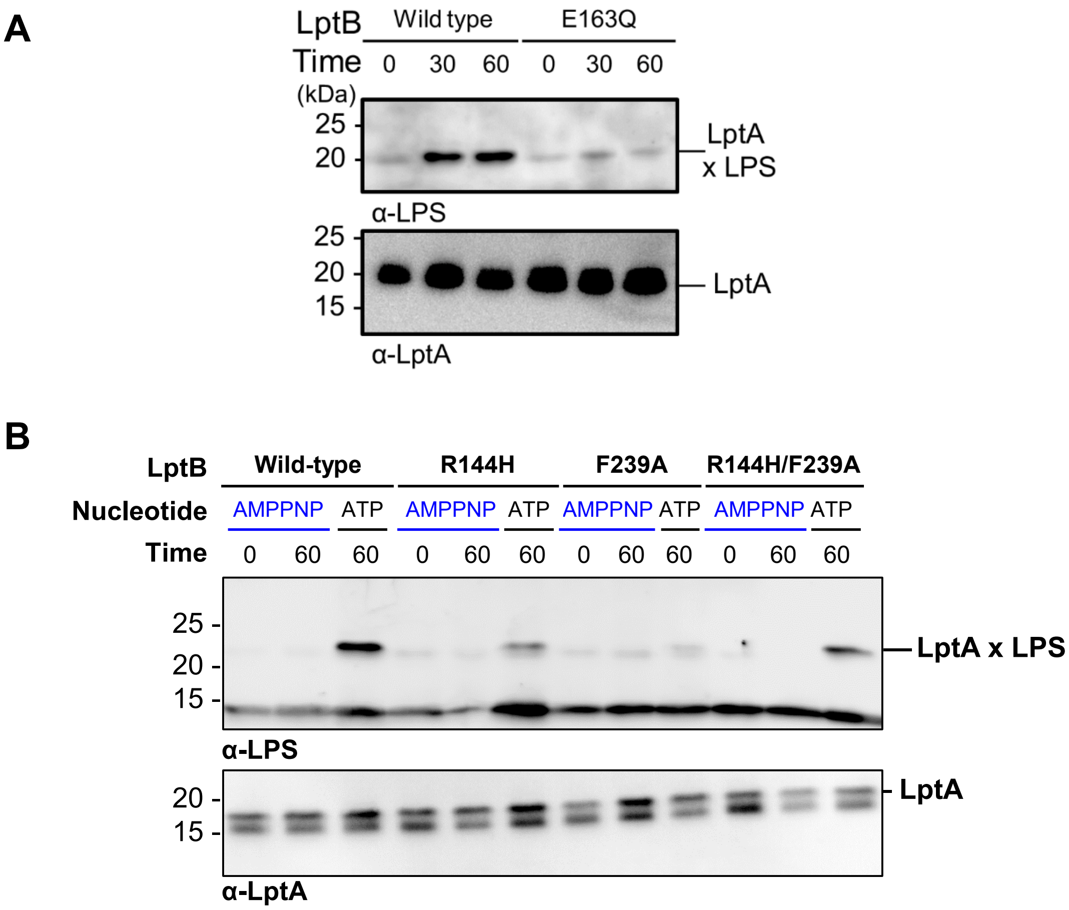

Figure S7: ATP hydrolysis is required for LPS release from proteoliposomes. **(A)** Complexes containing the catalytically dead $LptB^{E163Q}$ variant are defective in extracting LPS from liposomes. Assay monitoring LPS release from liposomes containing $LptB_2FGC$ complexes with $LptB^{WT}$ and catalytically dead $LptB^{E163Q}$ variants. Released LPS was UV-crosslinked to $LptA^{I36pBpa}$ and the resulting $LptA^{I36pBpa}$ x LPS adducts were detected by LPS immunoblot (top panel). Assays were initiated by the addition of ATP and incubated at 30°C for the indicated amount of time prior to crosslinking. LptA immunoblots are shown to confirm total LptA levels (bottom panel). Data are representative of three independent biological replicates. **(B)** *In vitro* assays for release of LPS from proteoliposomes containing $LptB_2CFG$ complexes with different LptB variants were performed in the presence of either ATP or non-hydrolysable AMPPNP. After 60 min, LPS was cross-linked to $LptA^{I36pBpa}$ and the resulting LPS-$LptA^{I36pBpa}$ adducts (marked LptA x LPS) were detected by LPS immunoblot (top panel). Total levels of $LptA^{I36pBpa}$ in the assay were confirmed by LptA immunoblotting (bottom panel). We also did not detect LPS transport by complexes containing $LptB^{WT}$ or $LptB^{R144H/F239A}$ when using the non-hydrolysable ATP analogs α,β-methylene ATP (ApCpp), 2-chloroadenosine β, γ-methylene ATP (AppCp), ATP-γS, and $AppNH_2$.
