# Supplementary material for: Combining Mutations That Inhibit Two Distinct Steps of the ATP Hydrolysis Cycle Restores Wild-Type Function in the Lipopolysaccharide Transporter and Shows that ATP Binding Triggers Transport
Source: mBio. 2019 Aug 20;10(4):e01931-19. doi: 10.1128/mBio.01931-19 (PMC6703430; doi:10.1128/mBio.01931-19)
Supplement: FIG S6 [file mBio.01931-19-sf006.pdf]

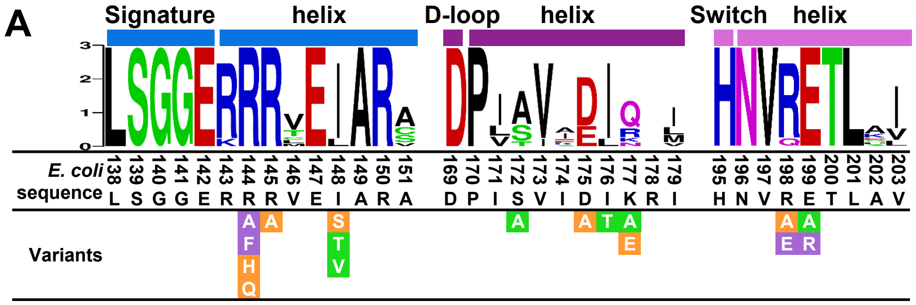

B

	Complements Δ*lptB*?			
	pET23/42LptB		pET23/42LptB-EHis$_8$	
	LB	Min	LB	Min
lptB(R144H)	NO	YES	YES	YES
lptB(R144Q)	NO	YES	YES	YES
lptB(R145A)	NO	YES	NO	NO
lptB(I148S)	NO	YES	YES	YES
lptB(D175A)	YES	YES	NO	NO
lptB(K177E)	YES	YES	NO	NO
lptB(R198A)	YES	YES	NO	YES
lptB(R198E)	NO	NO	NO	NO
lptB(E199A)	YES	YES	NO	YES
lptB(E199R)	NO	NO	NO	NO

C

	Zone of inhibition			
lptB allele	Bacitracin	Novobiocin	Erythromycin	Rifampin
lptB$^+$	(8)	(8)	8(10)	9(10)
lptB(I148T)	<6	<6	9(13)	11
lptB(I148V)	(9)	(8)	(10)	11
lptB(S172A)	(9)	(9)	(12)	10
lptB(D175A)	(8)	11(14)	9(12)	9
lptB(I176T)	<6	(9)	(13)	11
lptB(K177A)	(9)	<6	(12)	10
lptB(K177E)	7(9)	(9)	(15)	11
lptB(R198A)	15	15(24)	15(20)	13
lptB-EHis8	13	14(22)	16(20)	13
lptB(R144H)-EHis8	11	(9)	(16)	13
lptB(R144Q)-EHis8	30	(15)	(34)	34
lptB(I148S)-EHis8	9	(9)	(9)	12
lptB(I148T)-EHis8	(8)	(8)	(13)	10
lptB(I148V)-EHis8	(9)	(9)	(12)	10
lptB(S172A)-EHis8	(7)	(10)	11(13)	10
lptB(I176T)-EHis8	<6	<6	(13)	10
lptB(K177A)-EHis8	(7)	11(18)	9(12)	11

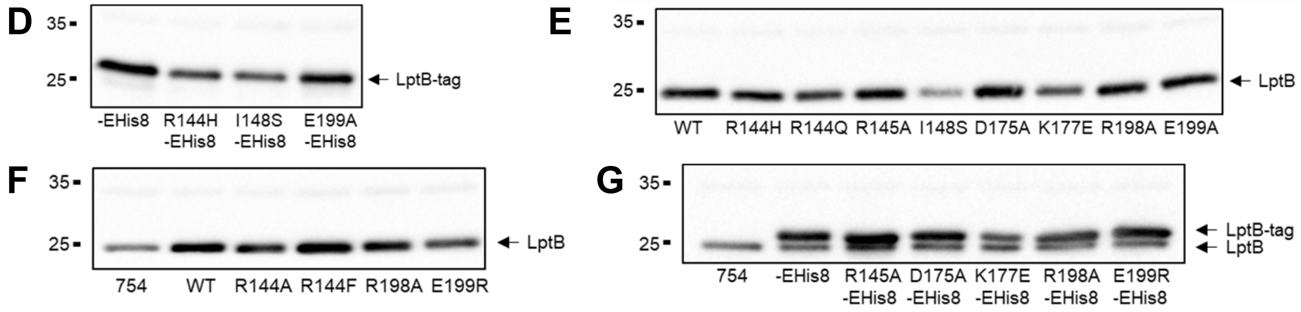

Figure S6: Synergistic effects of mutations combined with *lptB-EHis8* identifies a network of functionally-related residues. (A) Sequence logo generated, as described in Fig. 1D and Methods, of the conservation of residues in the signature, D-loop and switch helices (locations of each marked with thick lines). Below the logo, the sequence of the *E. coli* LptB^{WT} is shown in black and a summary of the functional analysis of single amino-acid substitutions to disrupt observed interactions between R144 and the CTD. Substitutions in pET23/42-LptB conferring no defects in strains carrying a chromosomal Δ*lptB* deletion are shown in green, those conferring partial loss of function are shown in orange, and those resulting in a total loss of function are shown in purple. **(B)** Ability of mutations that alter the indicated residue either in pET23/42LptB or pET23/42LptB-EHis$_8$ to complement a Δ*lptB* allele in the indicated media as described in Fig. S1A. **(C)** OM permeability of haploid strains carrying *lptB* mutations that exhibit synergistic effects with *lptB-EHis$_8$*. Disc diffusion assays were performed on LB plates. Numbers indicate diameter (in mm) of the zone of total growth inhibition or that of partial growth (in parenthesis). **(D)** LptB immunoblot of haploid *lptB-EHis$_8$* strains carrying pET23/42-LptB-EHis$_8$ grown in LB. **(E)** LptB immunoblot of samples from haploid *lptB* strains carrying pET23/42-LptB grown in minimal medium to allow growth of strains carrying conditionally lethal alleles. WT refers to NR2101, which produces LptB^{WT} from pET23/42-LptB. **(F)** LptB immunoblot of samples from merodiploid strains carrying the wild-type *lptB* allele and mutant *lptB* alleles encoded in pET23/42-LptB grown in LB. 754 refers to haploid strain NR754, the wild-type strain with chromosomal *lptB*$^+$; WT to NR2583, which produces LptB^{WT} from both the chromosome and pET23/42-LptB. **(G)** LptB immunoblot of samples from merodiploid strains carrying the chromosomal wild-type *lptB* allele and mutant *lptB-EHis$_8$* alleles encoded in pET23/42-LptB-EHis$_8$ grown in LB. 754 refers to haploid strain NR754; WT refers to NR1872, which produces LptB^{WT} from the chromosome and LptB-EHis$_8$ from pET23/42-LptB-EHis$_8$. Data are representative of at least three independent experiments.
